# BeetleBot: An integrated bioinspired soft robot for multimodal sensing and adaptive interaction

**DOI:** 10.1126/sciadv.aeg8849

**Published:** 2026-08-19

**Authors:** Inho Kim, Dickson R. Yao, Shukun Yin, Phuoc Thanh Tran-Ngoc, Jihong Min, Nathan Ng, Wenzheng Heng, Jiahong Li, Behnam Sadri, Hirotaka Sato, Sang Ouk Kim, Wei Gao

**Affiliations:** ^1^ Andrew and Peggy Cherng Department of Medical Engineering, Division of Engineering and Applied Science, California Institute of Technology, Pasadena, CA, USA.; ^2^ KAIST Institute for Nanocentury, Department of Materials Science and Engineering, Korea Advanced Institute of Science and Technology (KAIST), Daejeon, Republic of Korea.; ^3^ School of Mechanical & Aerospace Engineering, Nanyang Technological University, Singapore, Singapore.

## Abstract

Biological organisms exhibit tightly integrated motor functions, sensory input, and control systems that enable adaptive behaviors across diverse environments. Emulating such coordination in robotics remains challenging, as most soft machines compartmentalize actuation, sensing, and control. Here, we present BeetleBot, an adaptive beetle-bioinspired soft robot platform that seamlessly integrates programmable locomotion, object manipulation, sensory feedback, and intuitive human-machine interaction. BeetleBot features electrothermally actuated liquid crystal elastomer muscles embedded in modular leg structures and a soft gripper, with a flexible circuit enabling independent control. A wearable strain-sensing patch supports wireless gesture-based operation, while embedded vision and tactile sensors provide real-time environmental feedback. All functional components are fabricated through scalable additive manufacturing techniques. We demonstrate terrain navigation, object recognition, and gesture-control synchronized bidirectional interaction between the user and the robot. This multifunctional platform emulates the adaptability of living systems and establishes a promising foundation for next-generation intelligent soft robotics.

## INTRODUCTION

Biological organisms have evolved sophisticated strategies to adapt and respond to dynamic and unstructured environments (*1*–*3*). Their adaptability arises from the seamless integration of compliant structures, multimodal locomotion, sensory perception, and closed-loop control systems, allowing them to perform complex tasks such as navigation, object manipulation, and environmental sensing with high efficiency. Inspired by these capabilities, researchers have developed a wide range of biologically inspired robotic systems that mimic various animal functions (*4*–*13*)—ranging from turtle-like swimming (*14*), gecko-inspired climbing (*15*), and insect-mimicking flight (*16*) to octopus-modeled adaptability (*17*)—to operate in challenging environments. However, replicating the tightly coordinated, multifunctional adaptability of living organisms remains a central challenge in soft robotics.

A key limitation of current bioinspired robots lies in their reliance on bulky mechanical actuators, rigid components, and external control systems, which compromise mobility, energy efficiency, and system integration. In contrast, soft actuators composed of deformable, stimuli-responsive materials have emerged as a promising alternative (*18*, *19*). These materials enable safe, adaptive interactions with humans as well as environments while simplifying mechanical design and supporting scalable fabrication. Among these, electrothermally actuated liquid crystal elastomers (LCEs) offer programmable deformation, high work density, and compatibility with additive manufacturing (*20*–*27*). To achieve practical and intelligent soft robotic systems, it is essential to go beyond actuation and integrate sensing, control, and real-time human-machine interfacing within a compact and robust architecture. Such integration is critical for advancing soft robotics toward both autonomous operation and user-guided adaptability, enabling systems that can function independently while responding to external cues during collaborative or context-aware tasks. Advances in soft robotics have demonstrated enhanced functionality and individual performance metrics, yet few platforms achieve lifelike real-time bidirectional human-robot interaction integrating synchronized locomotion and multimodal sensory feedback within a single system (table S1) (*28*–*35*).

**Fig. 1.**
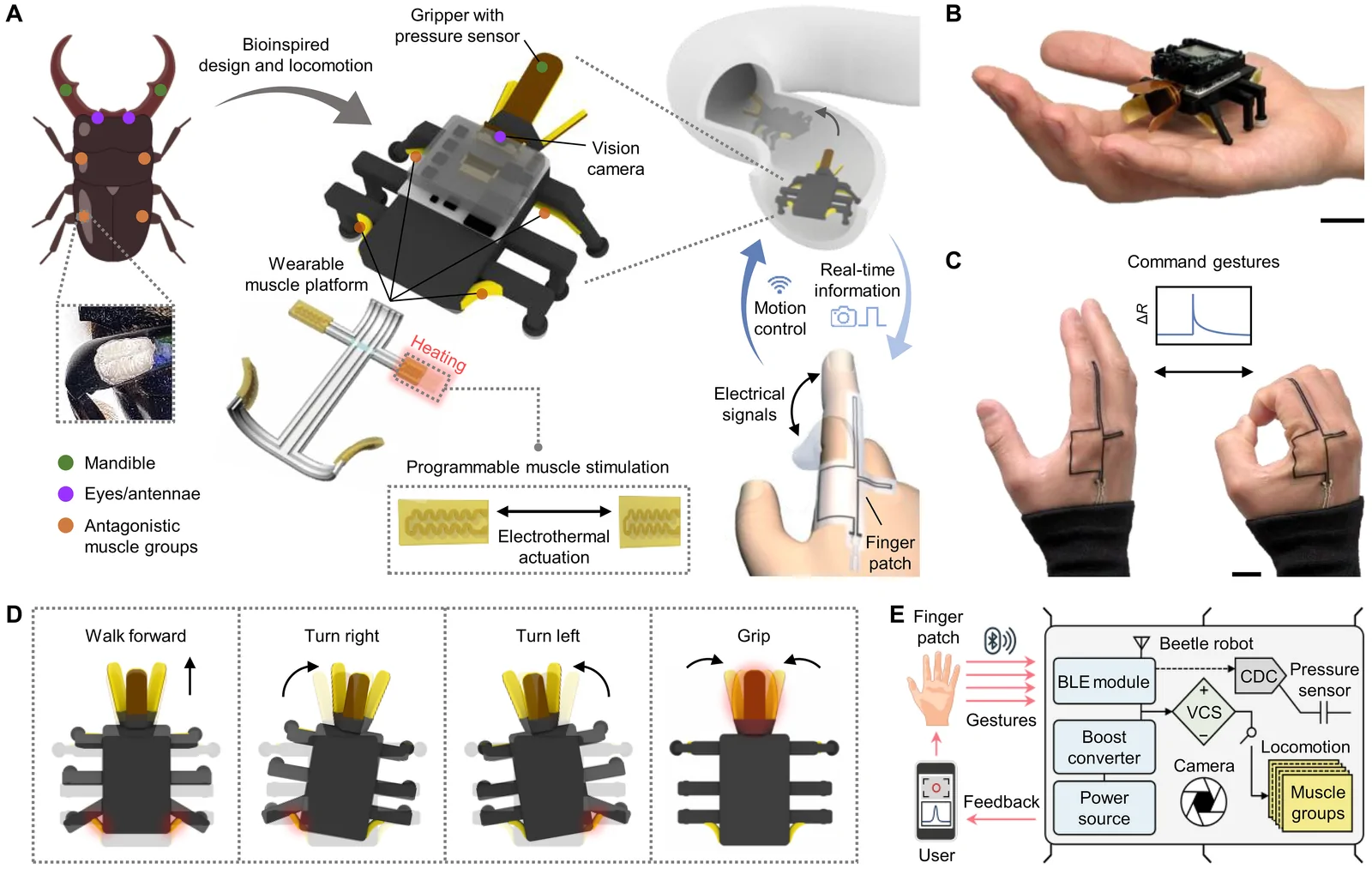
BeetleBot: An integrated bioinspired multifunctional soft robotic system. (**A**) Schematic overview of BeetleBot, a beetle-inspired soft robot that integrates bioinspired morphology, electrothermally actuated multimodal locomotion, embedded sensing, and an intuitive gesture-based human-machine interface. (**B**) Photograph of the assembled BeetleBot, featuring modular artificial muscle actuators, a thermally actuated soft gripper with capacitive tactile sensing, and an onboard visual camera. Scale bar, 2 cm. (**C**) Photograph of the wireless gesture control system, where user finger motions are detected via a wearable strain-sensing patch. Scale bar, 2 cm. (**D**) Schematic illustration of representative robot locomotion sequences, including forward movement and turning, driven by programmable actuation of leg muscle groups. (**E**) System-level diagram of the BeetleBot, detailing the signal flow from the user through the wearable control patch front end, the BLE command link, and the soft robotic components including camera, gripper, and locomotion units. VCS, voltage control system; CDC, capacitance-to-digital converter.

To address these challenges, we report BeetleBot, an integrated, bioinspired soft robotic system that replicates both the structural and functional attributes of beetles (Fig. 1A). Drawing on detailed biological analyses of beetle musculoskeletal architecture and gait mechanics, BeetleBot incorporates spatially programmable, electrothermally actuated LCE muscles embedded in modular leg structures and a mandible-like soft gripper for object manipulation. A printed flexible circuit independently controls these artificial muscles under low operating voltages (<5 V), enabling mechanically simple yet functionally rich actuation. The modular platform further integrates antenna-inspired tactile sensors, an eye-mimicking onboard camera, and multifunctional leg architectures, mimicking the sensory and locomotor capabilities of beetles. By replacing traditional motors and mechanical linkages with electrothermally driven soft actuators, BeetleBot achieves programmable lifelike motion while minimizing system complexity and enhancing adaptability in unstructured environments (Fig. 1B).

The robot is controlled via a wearable finger patch, whose strain signals are acquired through a tethered front end, classified into discrete commands, and relayed to the robot over a Bluetooth Low Energy (BLE) link (Fig. 1C). This allows the user to trigger locomotion modes including forward walking, turning, and object gripping (Fig. 1D). Simultaneously, embedded onboard vision and pressure sensors provide continuous real-time feedback to the user through a closed-loop interface, facilitating responsive and adaptive robot behavior during remote operation in confined or hazardous environments (Fig. 1E).

All functional components—including actuators, sensors, and circuitry—are fabricated using scalable additive manufacturing techniques such as direct ink writing (DIW) and laser cutting (fig. S1). This material-centric approach reduces mechanical complexity, facilitates miniaturization, and supports modular repair and customization. Comparable in size to a real beetle, BeetleBot integrates bioinspired design, soft actuation, embedded multimodal sensing, and wireless human-machine interaction into a single, compact platform. Together, BeetleBot demonstrates robust, synchronized behavior and real-time bidirectional interaction with a human operator, supporting autonomous navigation, object recognition, and task execution. This platform establishes a promising foundation for the next generation of intelligent soft robotics with potential applications in biomedical assistance, environmental exploration, and remote manipulation.

## RESULTS

### Fabrication and characterization of 3D-printed artificial muscle platform

**Fig. 2.**
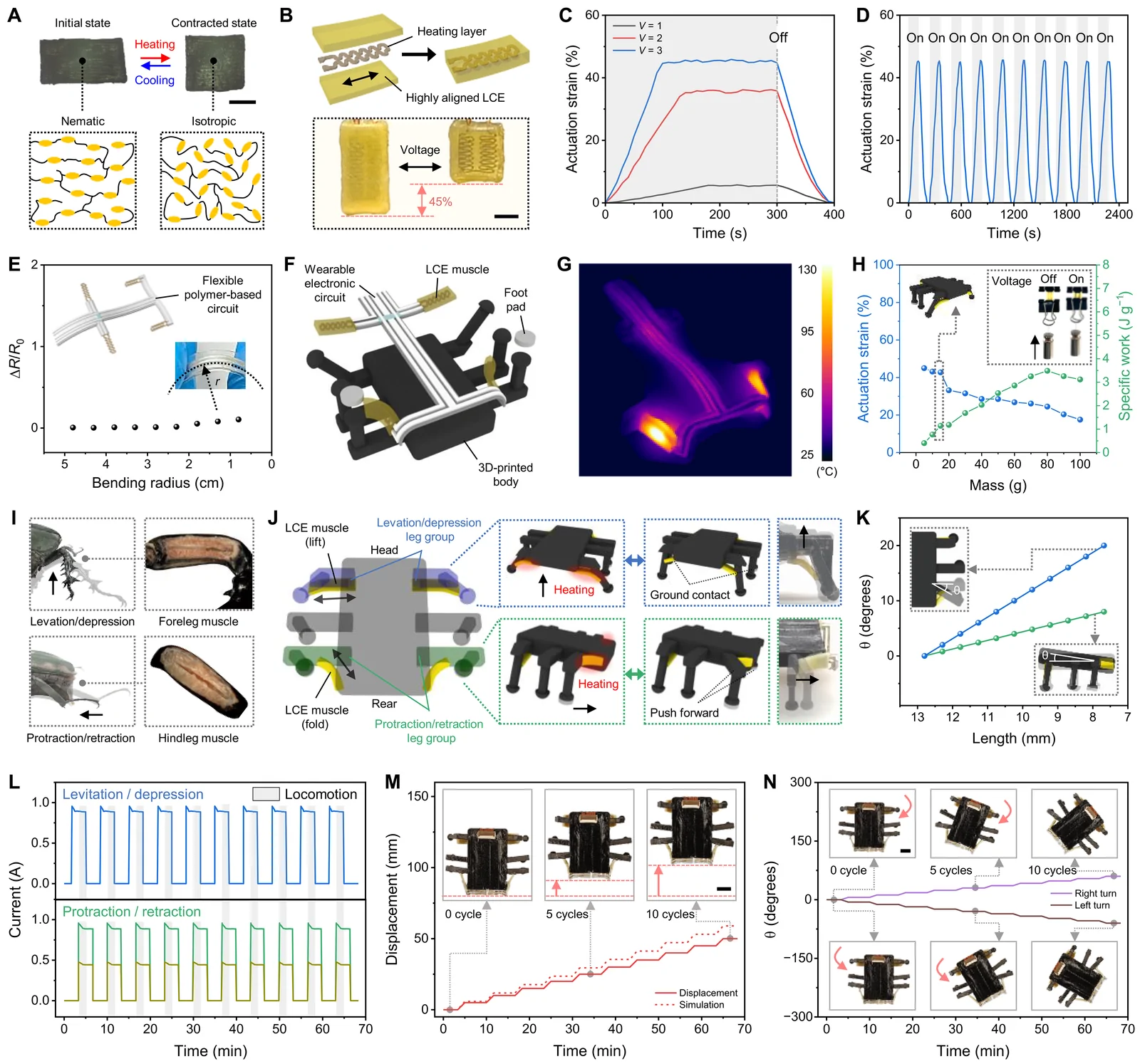
Bioinspired robot design and locomotion enabled by a wearable soft muscle platform. (**A**) Optical microscopy image of an LCE actuator and schematic of liquid crystal domain alignment during actuation-induced shape deformation. Scale bar, 3 mm. (**B**) Artificial muscle module composed of laser-patterned heating elements sandwiched between printed LCE layers. Scale bar, 3 mm. (**C**) Actuation strain of the LCE muscle module over time under varying applied voltages (1 to 3 V). (**D**) Reversible electrothermal actuation performance over 10 repeated on/off cycles upon electrical stimulation. (**E**) Mechanical flexibility and electrical properties of the integrated polymeric circuit with 70 wt % silver-SBS ink for programmable muscle actuation. (**F**) Schematic of the robot body assembly showing integration of the wearable muscle platform with a 3D-printed structural frame. (**G**) Thermal imaging showing spatially localized heating during selective muscle activation. (**H**) Maximum contraction strain and specific work capacity of LCE muscle under varying loads. (**I**) Photographs and anatomical illustrations of the musculoskeletal muscle groups in beetle legs. (**J**) Bioinspired anterior and posterior robotic leg groups with distinct muscle arrangements for levation/depression and protraction/retraction functions. (**K**) Voltage-dependent bending behavior of the robot leg segments replicating the angular displacement observed in biological beetle joints. (**L**) Measured current during repeated actuation of antagonist muscle pairs. (**M** and **N**) Linear displacement and angular turning trajectories resulting from multicycle muscle activation patterns. Scale bars, 1 cm.

Artificial muscles for the BeetleBot were fabricated using LCE-based actuators printed via DIW, which enables precise control over both liquid crystalline (LC) alignment and printed geometry (*36*–*39*). A viscoelastic LC oligomer served as the precursor, with its viscosity thermally tuned to allow continuous extrusion during printing (fig. S2). Shear forces and directional flow aligned the mesogen domains along the print path while simultaneously photocuring the spatially programmed nematic order (Fig. 2A and fig. S3). The resulting printed LCE actuators exhibited large, reversible, and anisotropic contraction along the mesogen alignment direction when heated above the nematic-isotropic transition temperature (*T*_ni_). An amine-acrylate LCE formulation was used to provide sufficient force output and mechanical robustness for stable long-term locomotion despite requiring a higher operating temperature than alternative low-transition-temperature LCE formulations.

Muscle actuation was achieved via Joule heating of laser-cut, flexible metallic wires embedded between the printed top and bottom LCE layers to form a laminated artificial muscle module (Fig. 2B). The serpentine-patterned heating element was designed as a structured metamaterial (*40*–*42*), with its geometry optimized for mechanical flexibility and reliable thermal actuation during the negative thermal expansion by LCE contraction (fig. S4). A wire thickness of 150 μm was selected to prevent burnout and cracking under compressive strain while maintaining efficient power consumption during operation (fig. S5). Heating currents above 0.45 A were sufficient to reach *T*_ni_ (∼110°C) for full contraction. The wire length varied across different muscle groups, leading to slight differences in the required voltage (ranging from 3 to 4 V) to achieve uniform heating.

For the actuator design, we optimized the actuator thickness by balancing load-bearing capability and thermal response speed within the thickness range capable of generating sufficiently large actuation strain (fig. S6). On the basis of this trade-off analysis, the actuator thickness was optimized to a 16-layer configuration to provide robust mechanical output for stable locomotion performance under the full robot weight.

Following the structural optimization, we systematically evaluated the electrothermal muscle actuation characteristics to assess its suitability as a mechanical component within the robotic system. Upon application of 3 V, the freestanding artificial muscle contracted by ∼45% of its original length and fully recovered after the voltage was removed (Fig. 2C). The actuator maintained consistent and repeatable strain output over more than 10 on-off voltage cycles after initial conditioning cycles, with negligible drift during subsequent operation (Fig. 2D and movie S1). In addition, the actuator demonstrated stable operation for more than 10 min and reached a peak actuation stress of 0.18 MPa (fig. S7)—within the typical range of mammalian skeletal muscle (0.1 to 0.35 MPa)—confirming its suitability for beetle-scale robotic motion (*43*–*45*). To optimize the actuator design for different robotic segments, we further assessed the influence of actuator length on electrical current, surface temperature, and mechanical displacement across a range of applied voltages (fig. S8). These measurements guided the selection of actuator parameters tailored to specific robotic body segments, ensuring precise movements without overheating.

To enable selective and programmable actuation of different muscle regions, we developed a fully 3D-printed wearable muscle platform using a flexible polymeric circuit composed of styrene-butadiene-styrene (SBS) and its conductive composites (Fig. 2E). Pure SBS served as the substrate, while silver-SBS composite ink with a filler ratio of 70 wt % was used for the conductive lines. The composition was optimized to balance electrical conductivity, stability, and viscoelasticity for reliable extrusion and consistent device performance (fig. S9). Each printed layer was additively fabricated under verified printing conditions to ensure reliable interconnection, while a silicone rubber precursor ink was printed as an electrical insulator between layers (fig. S10 and movie S2). Subsequently, the as-prepared heating elements were connected to both ends of the fully dried polymeric circuit. To prevent overheating during concurrent muscle activation, the ground line was printed with double thickness.

This circuit architecture minimized energy loss by thermally isolating the connection traces from the heating elements, allowing selective activation of target regions. For the whole wearable muscle platform, fabrication followed a layer-by-layer process: first printing the bottom LCE layer, then integrating the flexible circuit, and lastly printing the top LCE layer to complete the sandwich-structured actuator (movie S3). This sophisticated muscle platform demonstrated highly effective and independent actuation of muscle modules with consistent performance across multiple cycles (fig. S11 and movie S4).

### Beetle-inspired robot design for effective locomotion

The robot frame was fabricated using 3D-printed resin in the shape of a beetle, including six legs designed in desired geometry on the basis of LCE muscle actuation patterns (fig. S12). To enhance ground friction during locomotion and ensure proper interfacing with the wearable muscle, silicone rubber footpads were attached at predefined positions on the frame (Fig. 2F and fig. S13). Independent electrothermal actuation of the assembled robot was demonstrated by selectively applying voltage to different actuator regions (Fig. 2G). While local surface temperatures exceeded 100°C, infrared thermography revealed reliable, selective, and spatially localized heating of the active LCE muscle regions with minimal residual heat accumulation in the supporting robotic frame. This modular configuration allows the robot to execute multiple locomotion modes for diverse functional tasks. Notably, our robot design is compatible with scalable fabrication processes, allowing reproduction at various sizes through our standardized additive manufacturing techniques (fig. S14). The actuation performance and specific work capacity of the LCE muscles were evaluated under varying loads, including the total weight of the assembled robot with provisions for a gripper and onboard camera (Fig. 2H). These results confirmed that the artificial muscle modules provide sufficient output force to operate the entire robotic system.

To emulate efficient biological locomotion, we conducted a detailed anatomical and gait analysis of *Mecynorrhina torquata* beetles, which served as the basis for our robotic architecture (Fig. 2I). Insects use modular muscle groups to coordinate multidegree-of-freedom leg movements (*46*–*49*). We modeled this principle in our robot using artificial muscle modules arranged in antagonistic pairs to mimic levation/depression and protraction/retraction motions. As illustrated in Fig. 2J, the anterior leg group of BeetleBot is responsible for body lifting (levation/depression), while the posterior leg group provides forward propulsion (protraction/retraction), with both groups actuated independently using embedded muscle groups.

We performed quantitative analysis of beetle leg muscle behavior by measuring tibial angular displacement in response to electrical stimulation (fig. S15 and text S1). Through anatomical dissection and motion capture, we correlated muscle deformation with joint angles and derived a voltage-to-displacement relationship. This biological reference informed the specification of our LCE actuators. As shown in Fig. 2K, the robotic leg segments exhibited voltage-dependent angular displacement analogous to biological muscle motion, with anterior and posterior actuators achieving ∼20° and ∼12° of bending, respectively. This correspondence validates the fidelity of our bioinspired actuator design and highlights the potential of LCE-based actuators to replicate biological motion.

Using this leg configuration, we programmed coordinated activation of the two actuator groups to mimic the insect walking gait (*50*, *51*). As illustrated in fig. S16 and text S2, each walking cycle involved sequential activation of two antagonistic actuator groups: Heating the anterior actuators lifted the front of the body, tilting the rear section downward to establish ground contact of the posterior legs. Subsequent activation of the posterior actuators induced a folding motion that generated forward thrust. Deactivation of the anterior actuators then lifted the rear legs off the ground, followed by retraction of the legs to their initial configuration. The center of mass was designed to shift forward and backward during each tilting motion, enabling stable, dynamic locomotion.

To achieve continuous gait cycles, we implemented cyclic sequential activation of the leg groups, as shown in Fig. 2L. The shaded regions in the graph correspond to periods of locomotion, confirming that cyclic activation produced net displacement. Payload-carrying experiments further demonstrated that the robot maintained stable locomotion under additional loading conditions, although the locomotion speed gradually decreased with increasing payload because of the increased mechanical work required for body lifting and propulsion (fig. S17). The cumulative displacement profiles of the robot are presented in Fig. 2 (M and N). When both posterior leg actuators were actuated symmetrically, the robot advanced in a linear trajectory. In contrast, selective actuation of a single posterior leg group induced turning behavior. Repeated activation of a single side produced angular rotation, facilitating both left and right turns. Minor deviations in displacement and turning angle compared to simulation predictions are attributed to the mechanical constraints induced by the body mass and inertia of the robot during motions. The locomotion cycle was operated below the thermal cutoff frequency of the LCE actuator to ensure complete thermomechanical recovery during repeated operation.

### Soft gripper integrated with a pressure sensor

**Fig. 3.**
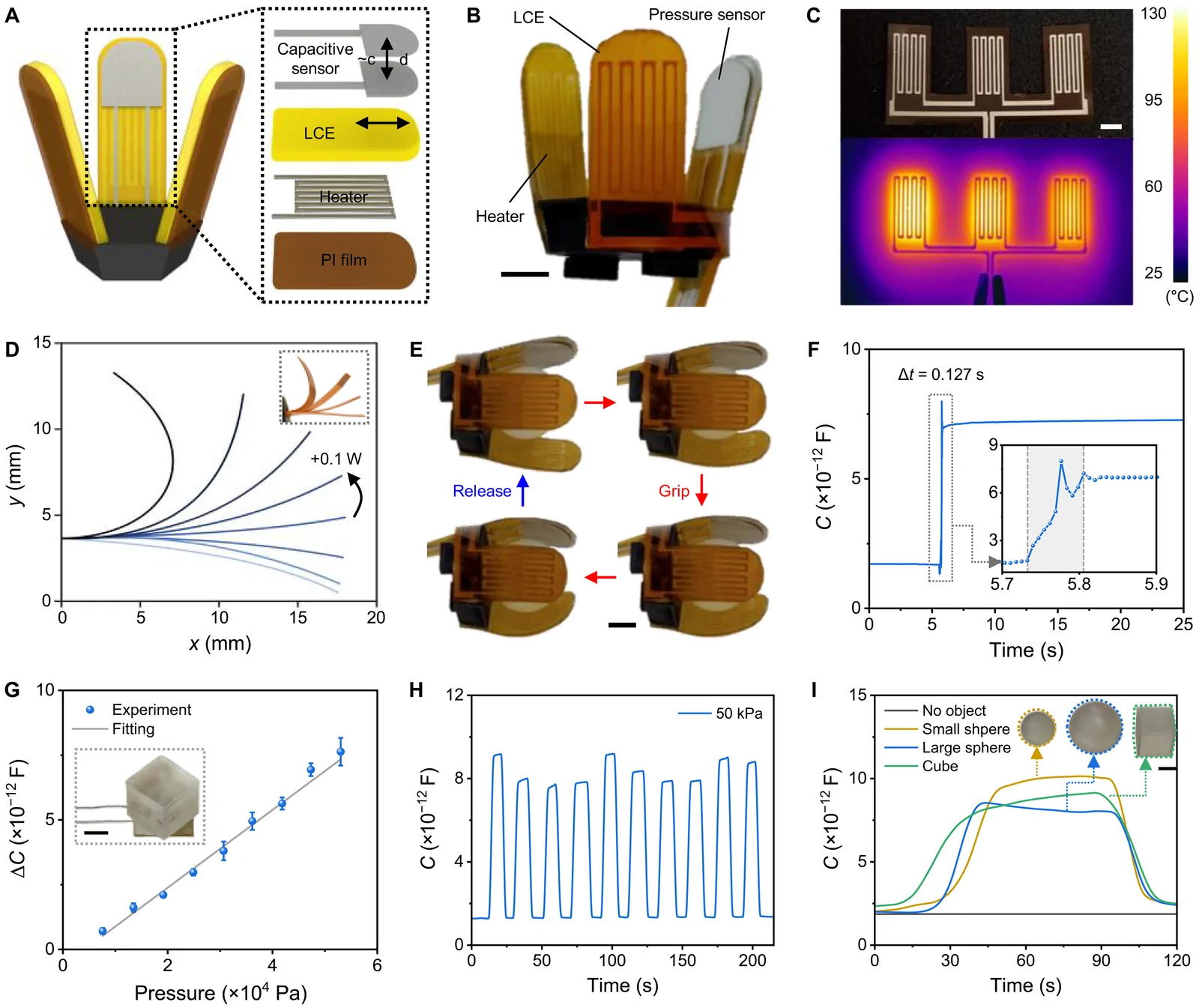
Soft gripper integrated with a capacitive pressure sensor for tactile object interaction. (**A**) Schematic of the multilayer soft gripper, composed of an LCE actuator, PI-based electrothermal heater, and an integrated capacitive pressure sensor. (**B**) Photograph of the gripper module mounted on the beetle-inspired robot head, incorporating embedded sensing functionality. Scale bar, 5 mm. (**C**) Infrared thermal imaging showing spatially localized heating within the LCE lamination zone during actuation. Scale bar, 5 mm. (**D**) Tip displacement of the LCE-PI bilayer structure under increasing voltage inputs. (**E**) Time-lapse images of reversible gripping and releasing motions. Scale bar, 5 mm. (**F**) Capacitance signal profile showing a rapid response upon contact with an object. (**G**) Pressure-capacitance calibration curve under varying loads. Scale bar, 3 mm. (**H**) Signal stability of the pressure sensor under repeated loading-unloading cycles. (**I**) Object recognition results based on size- and shape-dependent capacitance signals recorded during gripping of spheres and cubic objects. Scale bar, 4 mm.

To enable object manipulation with tactile sensing, we developed a soft gripper composed of a multilayered structure integrating a tactile sensor and an electrothermally actuated LCE muscle (Fig. 3A). The tactile sensor measures pressure through changes in capacitance resulting from variations in the distance between two conductive electrodes separated by an air gap (*52*, *53*). The actuator comprises an LCE muscle layer laminated with a flexible polyimide (PI) film with an electrothermal heater embedded (Fig. 3B). Infrared thermal imaging revealed rapid and localized temperature elevation across the heater and interconnect regions upon electrical stimulation, with surface temperatures approaching the *T*_ni_ of the LCE for effective shape deformation (Fig. 3C and fig. S18).

The bending behavior of the LCE actuator was characterized under varying heating powers. Tip displacement increased monotonically with input power, indicating a programmable and tunable bending response (Fig. 3D). This enabled reversible gripping and releasing actions, as illustrated in the time-sequenced frames shown in Fig. 3E.

The capacitive pressure sensor exhibited a rapid response time of 127 ms, with a representative signal characterized by a change in capacitance upon surface contact with an object, highlighting its suitability for real-time interaction and dynamic object interpretation (Fig. 3F). To quantify the sensing capability, we performed pressure-capacitance calibration under applied pressure loads. The sensor exhibited high sensitivity with a near-linear response up to 50 kPa (Fig. 3G). In addition, the sensor maintained a stable capacitive response over multiple loading-unloading cycles, confirming its mechanical durability and signal robustness (Fig. 3H). The sensor also maintained stable capacitance responses under elevated thermal conditions corresponding to the operating temperature range of the neighboring LCE actuator, indicating minimal thermal interference (fig. S19).

To assess the object recognition capability of the gripper, we tested it with samples of varying size and shape. As illustrated in Fig. 3I, the gripper successfully distinguished among different geometries on the basis of the amplitude and profile of the capacitance signal during gripping. This multimodal integration of soft actuation and pressure sensing establishes a functional platform for intelligent, feedback-driven manipulation in soft robotic systems.

### Development of strain sensor–based finger patch for wireless robot control

**Fig. 4.**
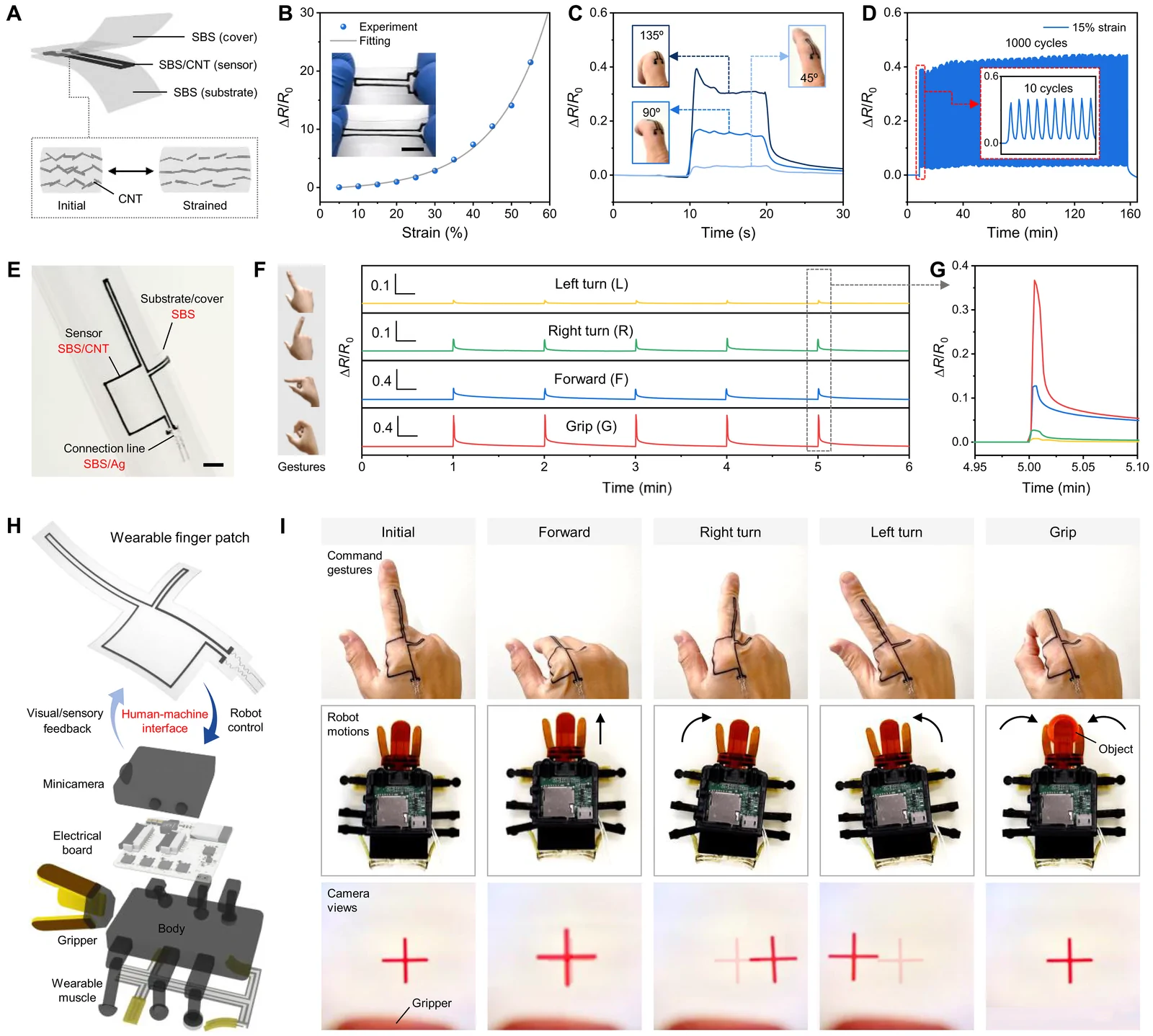
Gesture-based wearable control patch and synchronized human-robot interaction. (**A**) Schematic illustration of the structural design and working principle of the stretchable strain sensor based on a CNT-SBS nanocomposite. (**B**) Resistance response of the CNT-SBS strain sensor under increasing levels of mechanical strain. Scale bar, 1 cm. (**C**) Resistance variation measured at 45°, 90°, and 135° bending angles after sensor attachment to the user’s finger. (**D**) Durability test of the strain sensor over 1000 bending cycles at 15% strain. (**E**) Photograph of the wearable finger patch layout, featuring the integrated strain sensor and conductive interconnects. Scale bar, 1 cm. (**F**) Time-series resistance signals acquired from repetitive finger gestures used to classify control commands for the robot. (**G**) Magnified signals with discrete amplitude levels for reliable control inputs. (**H**) Schematic of the closed-loop user-robot interface, illustrating gesture-based control with real-time feedback, and an exploded 3D rendering of the modular BeetleBot assembly. (**I**) Real-time synchronization of gesture input, robot behavior, and visual feedback from the robot’s onboard camera. Each column shows a specific command (forward, right turn, left turn, and grip) with the corresponding robotic response and captured camera view.

To enable intuitive and remote control of the BeetleBot through human gestures, we developed a customized finger-mounted patch on the basis of stretchable carbon nanotube (CNT)-SBS nanocomposites. The strain sensor was optimized by tuning the CNT filler concentration and engineering the sensor structure. In this system, the conductive CNT network becomes disrupted under tensile strain, leading to strain-dependent resistance changes, as outlined in Fig. 4A (*54*–*58*).

To formulate printable inks, CNT-SBS composites were systematically evaluated for their electrical characteristics and shear-thinning rheological behaviors (fig. S20). For wearable sensor geometry optimization using multiple structural patterns under repeated finger bending conditions, the linear-shaped design showed stable and reproducible resistance responses suitable for direct sensing of human gestures (fig. S21). Subsequently, the influence of filler concentration was assessed, revealing that CNT contents below 5 wt % improved signal stability and sensitivity under cyclic bending. However, exceeding this threshold increased conductivity excessively, reducing sensitivity because of early signal saturation (fig. S22). The optimal formulation with 5 wt % CNT loading exhibited reliable stretchability and mechanical performance, validating its suitability as a wearable strain sensor (fig. S23). The sensor architecture comprised a single conductive layer encapsulated between two pure SBS layers used for both the substrate and top cover. This preserved mechanical resilience and ensured stable signal output during repeated deformation cycles, as the structural configuration substantially influences both signal amplitude and baseline consistency (fig. S24).

The sensor displayed a nonlinear yet monotonic resistance increase with strain, which was well described by an exponential model (Fig. 4B). Finite element analysis was used to visualize stress distribution throughout the strain sensor, revealing that as tensile strain increased, stress became progressively localized in the central conductive region, thereby supporting both the structural integrity and strain sensing capability of the design (fig. S25). When mounted on the user’s hand, the sensor reliably differentiated signal outputs across a range of bending angles (Fig. 4C and fig. S26). Long-term mechanical robustness was demonstrated over 1000 repeated bending cycles at 135° strain, during which the sensor maintained consistent performance with minimal signal drift (Fig. 4D).

### Assessment of synchronized human-robot interaction enabled by control and sensing

Building on the optimized sensor configuration, we redesigned the sensor layout to fit the finger geometry of the user and integrated conductive interconnects for real-time signal acquisition (Fig. 4E and movie S5). Resistance changes induced by different finger gestures were stably and reproducibly mapped to distinct command signals for wireless robot control (Fig. 4F and fig. S27). The time-series data from the wearable patch were translated into programmed actuation commands for robot locomotion. Four representative gestures including forward, left turn, right turn, and grip generated signal peaks with well-separated amplitudes, allowing for reliable classification and control inputs during repeated actions at various intervals (Fig. 4G and fig. S28).

By integrating the strain sensor with the soft robotic system, we implemented a seamless and functional human-machine interface capable of intuitive, gesture-based control over robotic motion, as demonstrated in Fig. 4H. A custom-designed circuit board enabled wireless communication and distributed power regulation across the system, supporting programmable and independent control of multiple artificial muscle modules, the soft gripper, and embedded capacitive sensors (fig. S29). As shown in Fig. 4I, each finger gesture successfully triggered a predefined robotic task, which was simultaneously verified through real-time visual feedback from the onboard camera module of the robot. The synchronization between human gesture input, robot behavior, and camera output confirms that our system can operate as an integrated, closed-loop interactive robotic platform with robust and intuitive control.

### Demonstration of interactive robotic task performance through closed-loop feedback

**Fig. 5.**
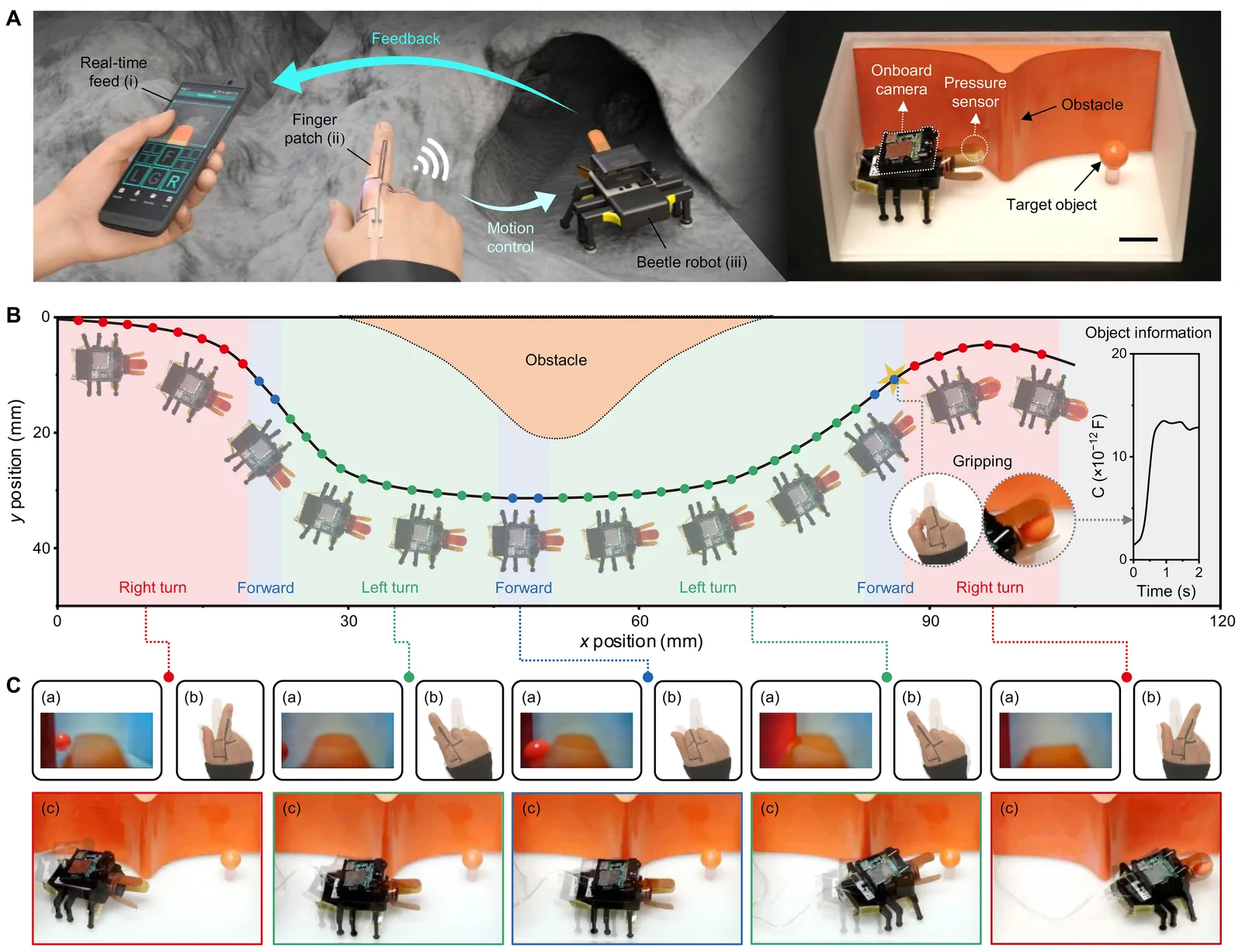
Demonstration of task execution through closed-loop human-robot interaction. (**A**) Schematic of an interactive robotic scenario. Left: The user controls the robot via a gesture-based finger patch while receiving real-time visual and tactile feedback. Right: Experimental setup showing a target object positioned beyond an obstacle, with the robot navigating the terrain to perform object manipulation. Inset: Image of the set of interactive robotic scenario. Scale bar, 2 cm. (**B**) Tracked trajectory of robot movement across eight motion segments demonstrating coordinated obstacle avoidance, target localization, gripping, and transport. Inset: Capacitance signal from the pressure sensor showing a sharp increase upon object contact, indicating successful detection and feedback transmission. (**C**) Sequential snapshots showing (a) onboard camera feedback, (b) user finger gestures, and (c) the corresponding robotic actions, illustrating synchronized operation between control input, visual feedback, and robot motion.

To validate the fully integrated functionality of our human-robot interface system, we conducted a task-oriented scenario involving remote navigation and object interaction. As illustrated in Fig. 5A, this demonstration combined real-time gesture-based locomotion control, obstacle avoidance, object detection, and sensory feedback as a substitute for direct human intervention.

The user wore a strain-sensing finger patch and issued gesture commands, which were wirelessly transmitted to the robot. Equipped with an onboard camera and a capacitive pressure sensor embedded in the soft gripper, the robot navigated a confined environment populated with obstacles and a target object. Throughout the task, continuous visual feedback was relayed to the user via a mobile interface. As shown in Fig. 5B and fig. S30, the robot followed a coordinated trajectory involving sequential right turns, left turns, forward locomotion, gripping, and object transport. A total of eight continuous motion segments enabled the robot to successfully avoid obstacles and reach the target object. The pressure sensor responded to contact with a distinct increase in capacitance, as shown in the inset graph, indicating successful detection of the spherical object and transmission of this information to the user (movie S6).

Time-resolved snapshots of the gesture commands and corresponding robot responses are presented in Fig. 5C and movie S7, highlighting the synchronized interplay between user input, robotic operation, and visual feedback. The system maintained stable, low-latency communication throughout the task, enabling the user to issue commands and adapt robot behavior in real time on the basis of both visual and tactile cues. This performance was enabled by the seamless integration of soft materials–based submodules designed for rapid actuation, sensing, and control.

Together, this closed-loop demonstration confirms our platform as an effective human-machine interface for remote manipulation of an artificial structure modeled after a real beetle through real-time decision-making and bidirectional communication in dynamic or unstructured environments.

## DISCUSSION

We have developed an integrated soft materials–based robotic system that replicates the structural and functional characteristics of beetles by uniting bioinspired architecture, multimodal actuation, embedded sensing, and an intuitive human-robot interface. Informed by detailed biological analyses of beetle musculoskeletal architecture and gait mechanics, we designed spatially programmable, electrothermally actuated LCE muscles, embedded within modular leg structures and independently controlled via a flexible printed circuit.

User-generated gestures, captured through a wearable strain-sensing finger patch, enable real-time wireless control of locomotion and object manipulation. Embedded visual and pressure sensors provide environmental feedback to establish a closed-loop, responsive system capable of operation in dynamic and unstructured environments. The material-driven modular design of our platform simplifies fabrication and supports scalable integration of multifunctional components.

Beyond demonstrating biomimetic mobility, this system offers a versatile framework for constructing high-performance, user-interactive soft robots. Its key features—including a wearable muscle interface, real-time responsiveness, lightweight construction, and robust wireless operation—position it as a foundational platform for next-generation intelligent soft robotics. Future generations of the platform could further improve practical applicability by incorporating alternative actuation mechanisms or engineering lower-transition-temperature actuator materials to enhance response speed and reduce thermal requirements for specific applications. This work lays the groundwork for future applications in medical robotics, environmental monitoring, and adaptive human-machine interaction.

## MATERIALS AND METHODS

### Materials

1,4-Bis[4-(6-acryloyloxyhexyloxy)benzoyloxy]-2-methylbenzene (RM82, Wilshire Technologies, 95%), *n*-butylamine (Sigma-Aldrich, 99.5%), Irgacure-369 (I-369, Sigma-Aldrich, 97%), SBS (Sigma-Aldrich, 30 wt % styrene), silver flakes (Sigma-Aldrich), toluene (Thermo Fisher Scientific), and multiwalled CNTs (Beijing Boyu Co.) were used as received without further purification. Ultraviolet (UV)–sensitive resin (Anycubic), silicone rubber (Smooth-On Inc.), silver conductive paint (SPI Supplies), copper film (DuPont Pyralux, AP8535R), PI film (McMaster-Carr), superglue (Krazy Glue), and medical tape (Adhesives Research) were used for device fabrication.

### Fabrication of BeetleBot

#### Preparation of the LCE muscle module

Serpentine-patterned heating elements were prepared by applying a cut-through method to copper-clad PI sheets (75-μm-thick PI with 18-μm copper on both sides) using a UV laser micromachining system (LPKF, ProtoLaser U4). The backside copper layer was selectively removed using the hatching feature for resistivity control. The resulting samples were ultrasonicated to remove debris from the ablated copper layer. Cross-linked LCE was prepared following the previously reported work (*20*, *28*). LC mesogen RM82 and cross-linker *n*-butylamine were added at a molar ratio of 1.1:1 in a 20-ml vial, followed by the addition of 2.5 wt % I-369 relative to the total LC mixture. The vial was vigorously vortexed under heating at 100°C to ensure uniform mixing and then placed in an oven at 80°C for 20 hours for step-growth polymerization while shielding the reaction from fluorescent light. The resultant yellowish LC oligomer was transferred into a 10-cm^3^ amber barrel and subsequently quenched in a freezer. DIW of the LC oligomer was performed using a three-axis motion control stage (Aerotech Inc.). The heating system (FM Keefe Company Inc.) included two heater coils of different diameters for the barrel and the nozzle tip, along with a temperature control console. The temperature was monitored using a *k*-type thermocouple to prevent the LC oligomer from exceeding *T*_ni_ during printing. The sample was then mounted onto the motion stage, set to a printing temperature of 60°C, and the ink was extruded through a 250-μm stainless steel nozzle under 551.581 kPa (80 psi) using a pressure-driven extrusion system (Nordson EFD, Ultimus V). The material was printed onto a glass substrate at a nozzle moving speed of 40 mm s^−1^ into the desired shape. The actuator geometry was designed in SolidWorks and converted into G-code using a silc3r software. During printing, the aligned oligomers were cross-linked into elastomers by exposure to a 360-nm UV lamp.

#### Preparation of the wearable muscle platform

The flexible polymeric circuit was fabricated using a DIW process in a layer-by-layer manner. All layers were designed in Autodesk and converted into G-code using a custom Python script. A 25 wt % SBS solution was prepared by dissolving SBS pellets in toluene via ultrasonication for 1 hour. To formulate a 70 wt % electrically conductive silver-SBS ink, silver flakes were homogeneously mixed into the SBS solution using a planetary centrifugal mixer for 5 min. Subsequently, the inks were loaded into a 10-cm^3^ clear barrel for printing. The pure SBS substrate layer was printed through a 200-μm tip at a moving speed of 4 mm s^−1^ under an extrusion pressure of 68.947 kPa (10 psi). Conductive traces were then printed using a silver-SBS composite ink through a 410-μm tip at a moving speed of 5 mm s^−1^ under an extrusion pressure of 34.473 kPa (5 psi). A silicone rubber layer was deposited at the appropriate position using a 250-μm tip at a moving speed of 1 mm s^−1^ under 41.368 kPa (6 psi) to serve as a dielectric barrier, providing electrical isolation between the layers. Each SBS-based layer was left to dry for more than 10 min to allow sufficient toluene evaporation and solidification before the next layer. Subsequently, as-prepared heating meshes are connected to each end of a flexible polymeric circuit by applying silver conductive paint and then sintered by heating. Last, two identical, previously described LCE muscles are printed on either side of the polymeric circuit, forming a sandwich-structured LCE muscle module. This assembly was then hot pressed at 50°C using a heat gun and subsequently exposed to UV light for an additional 10 min on each side. This process is repeated at four designated positions to form a complete wearable muscle platform with independent muscle modules.

#### Preparation of the gripper and pressure sensor

The heating circuit for the gripper was fabricated via DIW by patterning conductive silver paint onto a 75-μm-thick PI substrate. The concentration-adjusted silver paint was extruded through a 250-μm stainless steel nozzle at a moving speed of 10 mm s^−1^ under an extrusion pressure of 68.947 kPa (10 psi). A single layer was deposited for the heating regions, while three layers were applied to the interconnect lines to introduce a conductivity gradient. LCE actuators were fabricated using the same DIW process as previously described and laminated onto the PI layer to form PI-LCE bilayers with a 400-μm-thick active layer, ensuring optimal bending without delamination. Cyanoacrylate adhesive was applied at the ends of the LCE to secure it to the PI layer, away from the heating elements. Capacitive pressure sensor components were shaped by patterning a 12-μm-thick PI film with a CO_2_ (carbon dioxide) laser cutter (Universal Laser System) and applying a thin layer of conductive silver paint to one side. The sensors were assembled by laminating the silver-coated PI over an LCE actuator, with commercial tape added as a dielectric spacer, and aligning the top and bottom plates. One corner of the top plate was glued to the LCE, while the remaining edges were left unfixed to allow a large air gap for increased sensitivity. Excess PI and LCE were trimmed to define the final gripper shape.

#### Assembly of robotic modules

Three-dimensional (3D) robot frame models were designed using SolidWorks and fabricated via stereolithography (Elegoo Inc., Saturn 3). The printed molds were cleaned with isopropyl alcohol and cured under a 50-W, 360-nm UV lamp for 10 min. The joints and body components were assembled into a robot frame via interlocking hole-and-pillar structures. The as-prepared gripper module was attached to a hexagonal resin structure using cyanoacrylate adhesive as a head structure of the robot. Subsequently, the head and the wearable muscle platform were glued to the robot frame at predefined locations. The muscle and gripper flexible circuits were connected to a custom-designed circuit board and mounted on the dorsal side of the robot. A wireless minicamera (Rettru) was attached on the top of the robot. The robot was continuously powered by a power supply (KEITHLEY, 2231A-30-3) connected to the circuit board.

#### Integration of the robot electronic system

The electronic system of the beetle robot includes a voltage regulator (TPS610982, Texas Instruments) that converts the 3.4-V power supply output to a stable 3-V supply for the BLE module using a buck-boost topology to handle voltage variations above or below the target. Wireless commands are managed by an STM32WB5MM microcontroller (STMicroelectronics), while grip strength feedback is read through a capacitive sensor interface (FDC1004, Texas Instruments), enabling a closed-loop control of the soft gripper under wireless command. Gripper actuation is powered by an adjustable boost converter (TPS63070, Texas Instruments), and independent power for the leg actuators is provided by four adjustable voltage regulators (ADM7172, Analog Devices).

### Fabrication of wearable finger patch

The strain sensor–based finger patch was fabricated using the same DIW setup and design flow as the flexible polymeric circuit described above. First, the substrate layer was printed using a 25 wt % SBS solution with a 200-μm tip at a moving speed of 4 mm s^−1^ under an extrusion pressure of 68.947 kPa (10 psi). A 5 wt % CNT-SBS ink was prepared and printed using a 410-μm tip at a moving speed of 4 mm s^−1^ under an extrusion pressure of 124.106 kPa (18 psi). Interconnection lines were deposited using a 60 wt % silver-SBS ink through a 250-μm tip at a moving speed of 2 mm s^−1^ with an extrusion pressure of 206.843 kPa (30 psi). A final pure SBS layer was printed over the same dimensions and printing conditions as the substrate to encapsulate the structure. After the patch was completely dried, it was carefully peeled off from the glass substrate. A piece of medical tape was laser cut to match the shape of the patch and applied to the back side of the patch for conformal attachment to the user’s hand.

### Finger patch data acquisition and robot communication

Strain signals from the CNT-SBS sensor on the finger patch were routed through a flexible printed circuit to a voltage-divider front end on an Arduino Uno R3, which digitized the signal and streamed it to a host PC over USB serial. On the PC, a threshold-based classifier assigned each trace to one of four command classes (forward, left turn, right turn, and grip) on the basis of the discrete amplitude levels shown in Fig. 4G. The classified command was then transmitted wirelessly to the robot over a BLE link, where the on-board microcontroller drove the corresponding leg-muscle and gripper heating channels through the flexible polymeric circuit. The wearable patch was therefore tethered to the acquisition front end, while the robot operated untethered from the user during gesture-controlled locomotion.

### Biological study of beetles

Adult *M. torquata* beetles (Coleoptera, Kingdom of Beetle) were used for muscle displacement studies. Beetles were housed individually in 15 by 15 by 20–cm plastic containers and fed sugar jelly every 2 to 3 days under controlled conditions (25°C and 60% relative humidity). Only male beetles of 6 to 8 cm in length were selected. Animal use was approved by the Agri-Food and Veterinary Authority of Singapore (AVA; HS code: 01069000; product code: ALV002). Each beetle was anesthetized by placing it in a CO_2_-filled plastic bag for 1 min. Two silver electrodes (A-M Systems, 127 μm uninsulated, 178 μm with Teflon insulation) were implanted into the flexion muscle group responsible for tibial retraction. The beetle was fixed to a plastic frame using nylon cable ties. Holes of 0.5 mm in diameter were punctured in the cuticle with an insect pin (Indigo Instruments, no. 3) at predefined positions. The femur was secured to restrict movement to the tibia. The insulation was stripped from the electrode tips, and a metal stopper knot was soldered 2 mm from the tip to control the penetration depth. The electrodes were connected to a waveform generator (Keysight, 33500B Series), delivering square pulses with a 1-ms width at a 30-Hz frequency. The voltage started at 0.3 V and was increased in 0.3-V steps. Tibial angular displacement was recorded using a VICON motion capture system by tracking three reflective markers placed on the femur, joint, and tibia. Angles were calculated on the basis of the difference between the femur-joint and joint-tibia vectors. Muscle morphology was examined by dissecting the front leg. The cuticle was opened using microdissecting scissors and removed with fine tweezers. The foreleg was fixed with beeswax and imaged under a digital microscope (Keyence, VHX-6000). The tibia was manually rotated with tweezers, and nine images were captured at angles between 5.17° and 62.97°. Images were imported into SolidWorks to assess the relationship between tibial angle and muscle length. Muscle lengths were converted to millimeters using a calibrated scale bar from the microscope image. A two-step analysis was conducted: (i) Tibial angles from VICON data were correlated with muscle lengths from SolidWorks, and (ii) a curve fit was applied to model the relationship between the angle and muscle displacement. This enabled conversion of stimulation voltage to corresponding muscle displacement.

### Characterization techniques

The rheological properties of the customizable inks were analyzed using a rheometer (Anton Paar, MCR 30). Surface temperature profiles during actuation were captured with an infrared camera (FLIR T540). All the LCE muscle performances and robot locomotion behaviors were recorded using a digital camera (Canon 60D) and analyzed with ImageJ. Mechanical properties of strain sensors and actuators were tested with a universal testing machine (Instron 5965) with a 25-N load cell. During the measurements, the samples were fixed at both ends using clamps. Actuation stress was measured under a constant strain of 3%. Electromechanical properties of the strain sensors and finger patch were evaluated using an electrochemical workstation (CHI660E) to monitor real-time resistance changes. The capacitance of pressure sensors was measured using a Keithley instrument (Keithley 4200A). Finite element analysis was carried out using commercial software COMSOL Multiphysics to analyze stress distribution during deformation of CNT-SBS sensor lines under varying strain levels at 5, 10, and 15%. Tetrahedral elements with refined meshes allowed modeling of the stretching process with testified accuracy. Young’s moduli (*E*) of SBS and CNT-SBS were set to 12.9 and 18.5 MPa, respectively.
