# Supplementary material for: BeetleBot: An integrated bioinspired soft robot for multimodal sensing and adaptive interaction
Source: Sci Adv. 2026 Aug 19;12(34):eaeg8849. doi: 10.1126/sciadv.aeg8849 (PMC13488934; doi:10.1126/sciadv.aeg8849)
Supplement: Supplementary file 1 [file sciadv.aeg8849_sm.pdf]

Science Advances
AAAS

Supplementary Materials for

BeetleBot: An integrated bioinspired soft robot for multimodal sensing and adaptive interaction

Inho Kim *et al.*

Corresponding author: Sang Ouk Kim, sangouk.kim@kaist.ac.kr; Wei Gao, weigao@caltech.edu

Sci. Adv. **12**, eaeg8849 (2026)
DOI: 10.1126/sciadv.aeg8849

The PDF file includes:

Supplementary Text S1 and S2
Figs. S1 to S30
Table S1
Legends for movies S1 to S7

Other Supplementary Material for this manuscript includes the following:

Movies S1 to S7

Text S1. Biological study of beetles

To ensure that the robot's motion reflects real insect biomechanics, we conducted a quantitative characterization of leg muscle actuation in the front leg of *Mecynorrhina torquata* beetles. Specifically, the angular displacement of the tibia segments in living specimens was measured in response to varying electrical stimulation voltages. A motion capture system tracked tibial rotation using three reflective markers (**fig. S15A** to **C**), revealing a monotonic increase in angular displacement up to ~33° at 2.4 V (**fig. S15D**). This movement trajectory, visualized in x–y space (**fig. S15E**), was further used to correlate joint angle with internal muscle deformation. Anatomical dissection and digital image analysis of the foreleg revealed a direct relationship between tibia angle and flexor muscle length, allowing us to construct a quantitative mapping between angular displacement and muscle contraction (**fig. S15F**). Combining these datasets enabled derivation of a final input–output map from voltage to muscle displacement, which was used as a reference benchmark for our LCE actuator design (**fig. S15G**).

Text S2. Locomotion mechanism of bio-inspired robot

As shown in **fig. S16A**, the soft machine-legged robot completes a full walking cycle through the sequential activation of antagonistic actuator groups. The motion begins at phase 1 (P1), the initial stance phase, where the robot maintains a stable posture. At phase 2 (P2), contraction of the levation/depression muscles in the anterior leg group causes the body to tilt backward, inducing ground contact at the rear footpads. This ground engagement generates frictional forces, anchoring the posterior leg group for propulsion. During phase 3 (P3), contraction of the protraction/retraction muscles in the posterior leg group pushes the body forward or induces directional turning, depending on whether one or both protraction/retraction muscles are activated. In phase 4 (P4), the anterior leg group extends while the posterior leg group remains flexed, lifting the rear part of the robot and raising the posterior footpads off the ground, as the weight of the robot is biased toward the front due to the frame design, thereby completing the displacement. Finally, the posterior leg group returns to its original position, restoring the robot to the P1 configuration and completing one full gait cycle. The current output and displacement data for each LCE muscle group, shown in **fig. S16B**, confirm the coordination between muscle groups, highlighting consistent transitions between phases and validating the effectiveness of this biomimetic locomotion strategy. The levation/depression muscles (blue) maintained a constant current of approximately 1.1 A throughout the walking sequence, allowing the front legs to sustain ground clearance and body lift. Meanwhile, the protraction/retraction muscles (green and yellow) were selectively activated to modulate posterior leg group motion, exhibiting a stable ~0.55 A for single activation and ~1.1 A for dual activation, as illustrated by the horizontal line shifts between the green and yellow channels. The red plot in fig. S16B represents the spatial displacement of the robot at a designated reference point on the body during a single cycle. Displacement increased synchronously with posterior leg group locomotion during the propulsion phase, peaking at P3 before relaxing during P4. The coupling between current output and displacement response reflects effective thermomechanical actuation of the LCE muscles. Together, these results demonstrate that coordinated control of antagonistic actuator groups enables insect-inspired gait generation in soft robotic systems. Furthermore, the consistency and repeatability of the displacement data over multiple cycles validate the stability and robustness of LCE actuator performance.

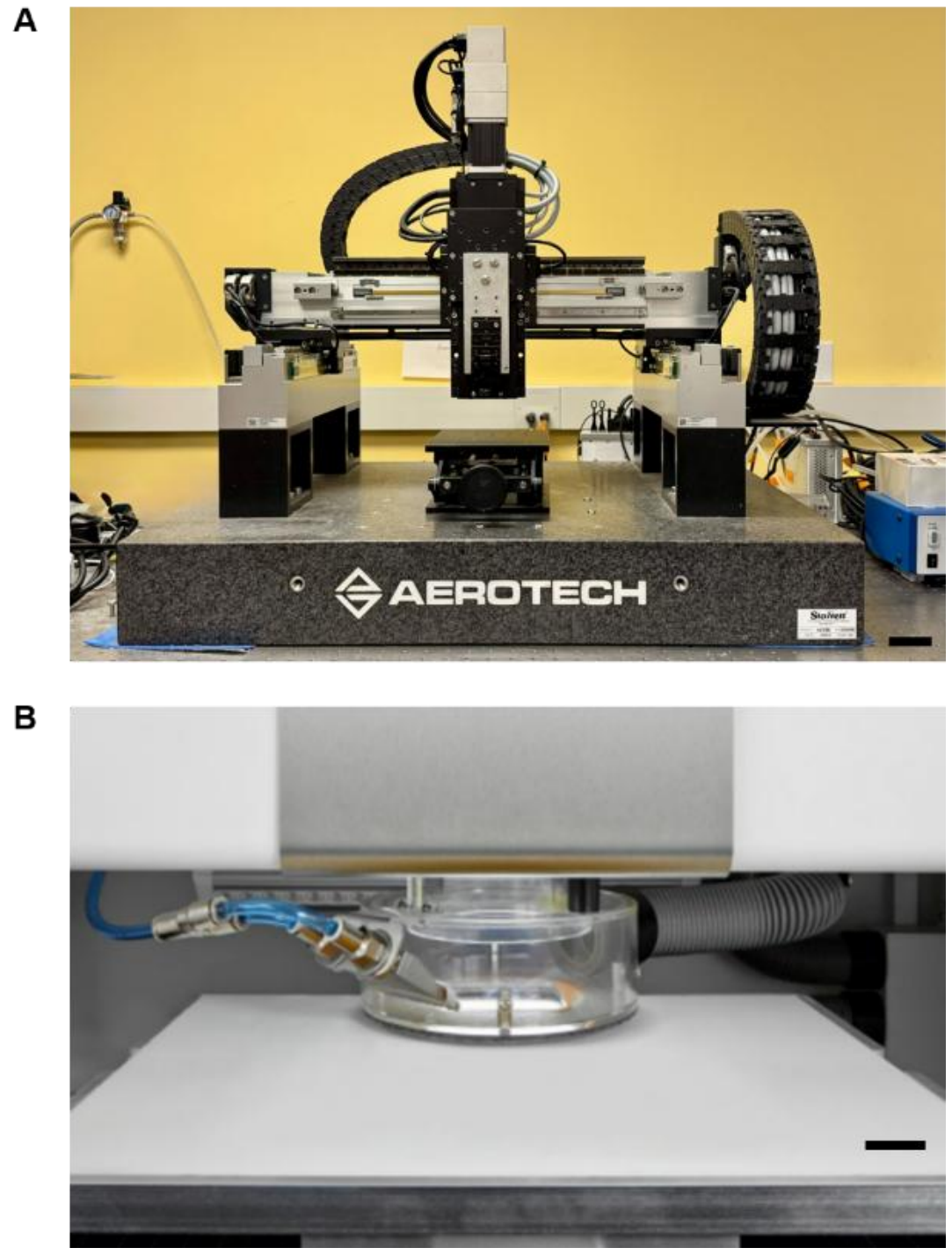

Fig. S1. Additive manufacturing systems for fabricating muscle and sensing modules. (**A**) Optical image of the direct ink writing (DIW) platform equipped with a multi-axis motion control stage used printing liquid crystal elastomer (LCE) actuators. Scale bar, 5 cm. (**B**) Optical image of ultraviolet (UV) laser micromachining system employed for high-precision patterning of flexible circuit components and sensor elements. Scale bar, 5 cm.

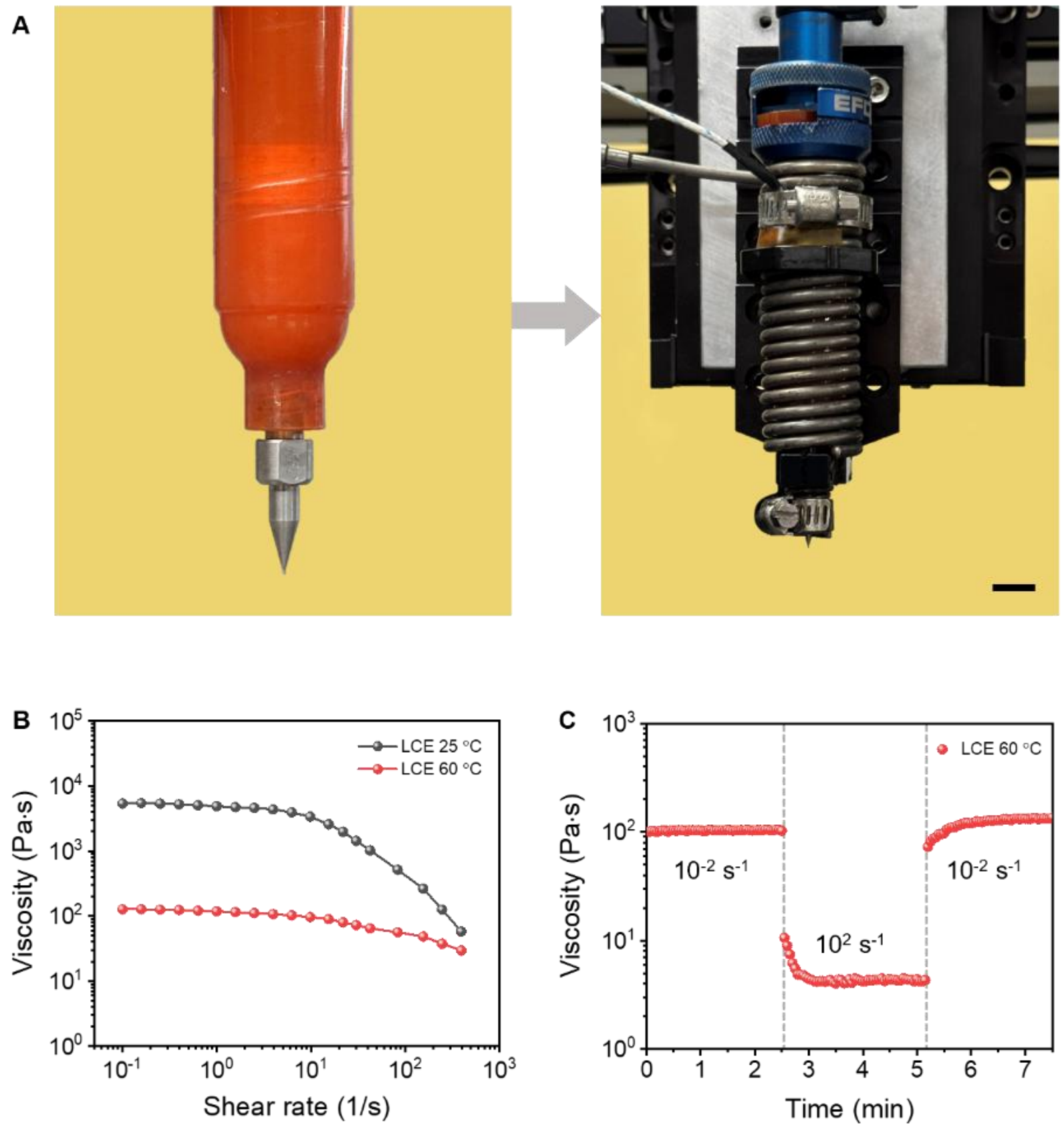

Fig. S2. Customized heating system for tuning ink rheology during DIW of LCE actuators. (**A**) Optical image of the temperature-controlled DIW printhead, with integrated barrel and nozzle heating to maintain uniform thermal conditions for LC oligomer extrusion. Scale bar, 1 cm. (**B**) Rheological profile of LC oligomer ink showing viscosity as a function of shear rate measured at room temperature (25 °C) and elevated printing temperature (60 °C). (**C**) Shear-thinning behavior of the ink demonstrated by Viscosity variation under different shear rates.

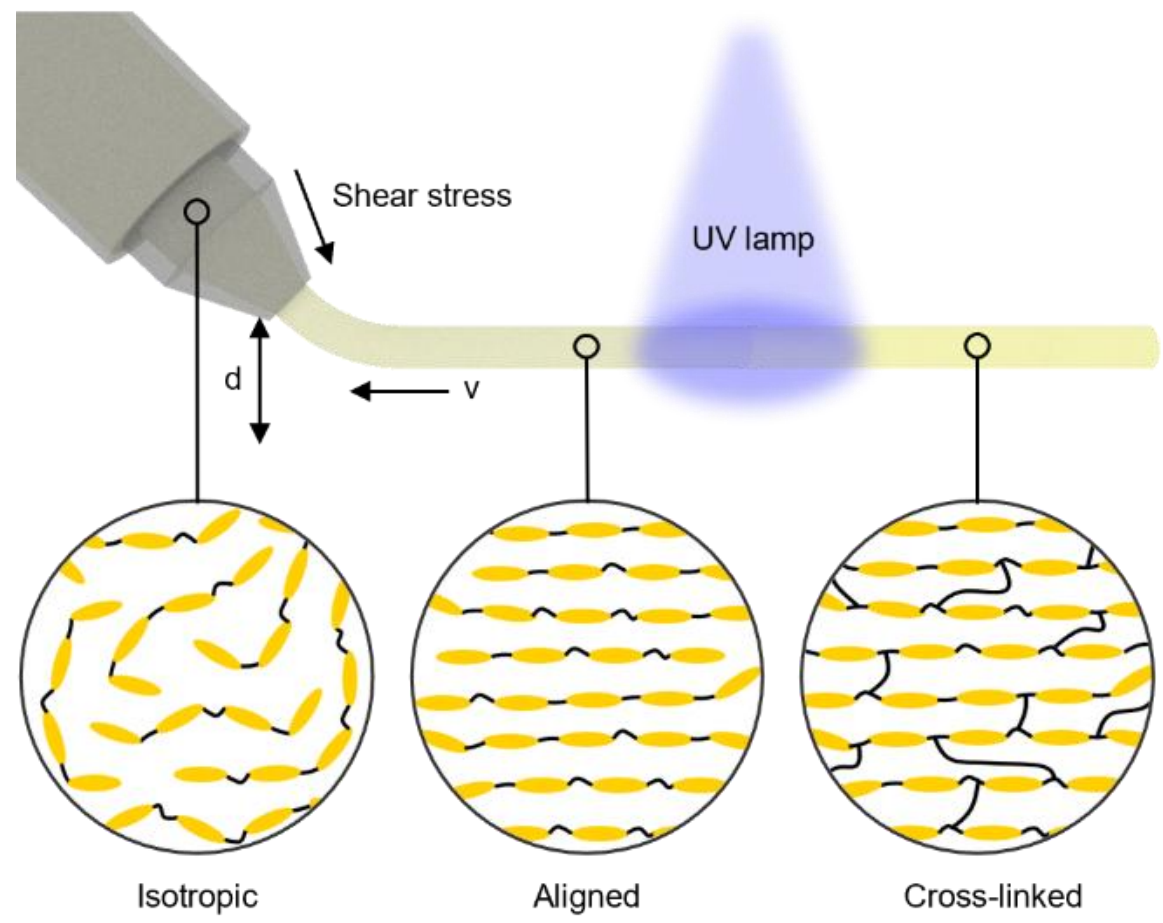

Fig. S3. Molecular dynamics of LC mesogens during the LCE DIW printing process. Schematic illustration of the printing process showing the evolution of liquid crystal (LC) mesogen orientation: initially disordered in the isotropic phase, mesogens align along the shear direction during extrusion through the nozzle, and are subsequently fixed in their aligned configuration via UV-induced crosslinking.

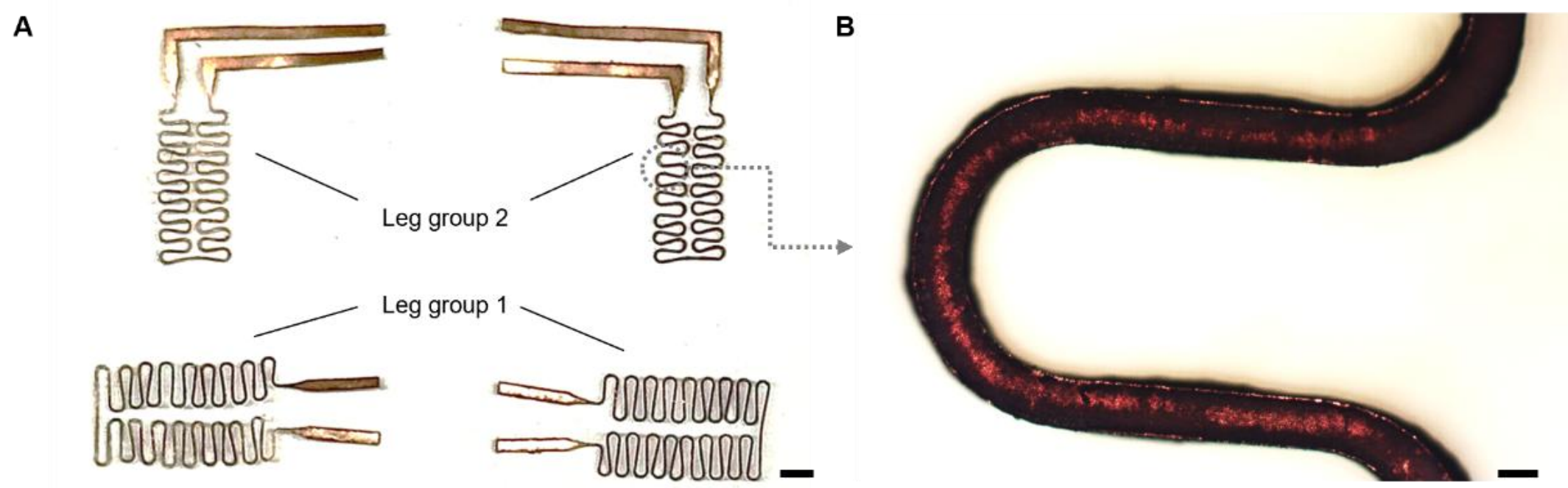

Fig. S4. Serpentine-patterned heating elements for electrothermal actuation of LCE muscle modules. (**A**) Two sets of laser-cut metallic wires corresponding to anterior and posterior leg actuators. Serpentine geometry enhances mechanical compliance and accommodates thermal expansion during repeated actuation cycles. Wire designs were optimized for symmetry and consistent actuation across leg modules. Scale bar, 3 mm. (**B**) Optical microscopy image of a 150 μm-thick copper wire. Scale bar, 100 μm.

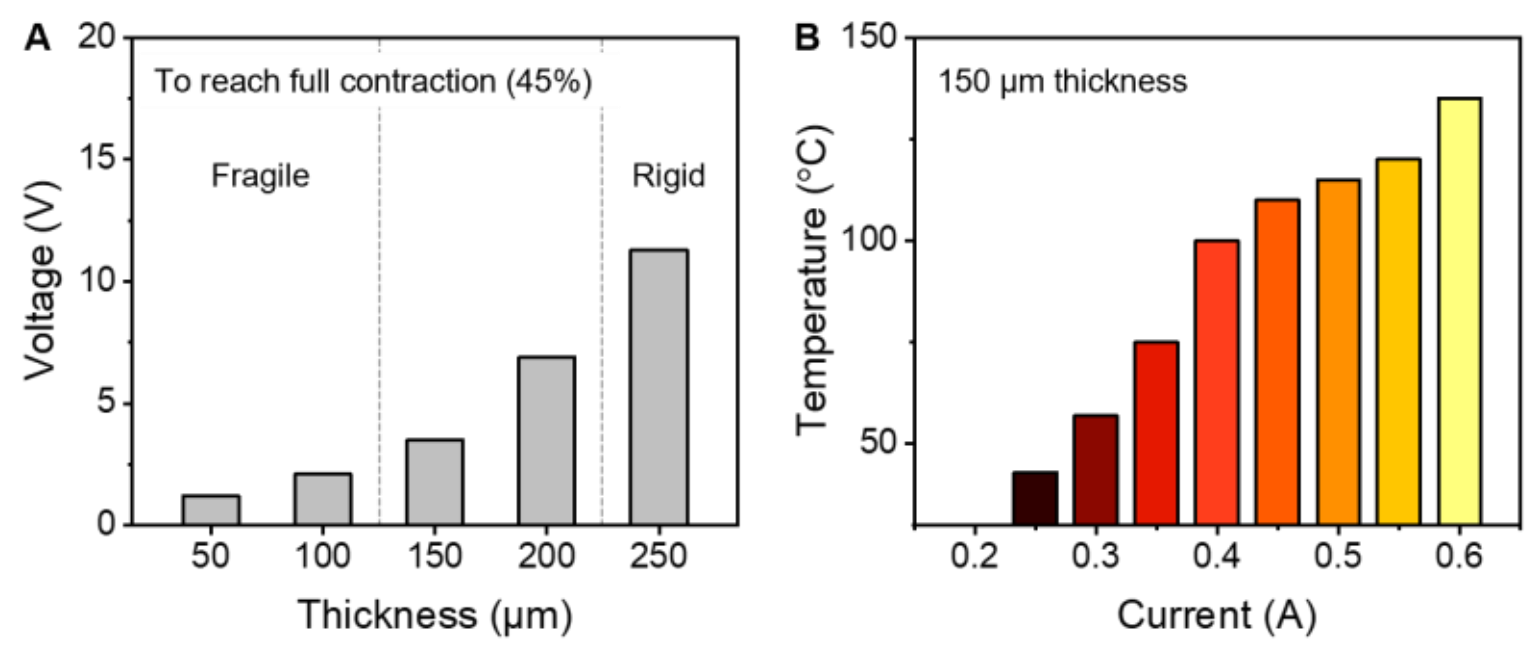

Fig. S5. Optimization of wire thickness for thermal and mechanical performance. (**A**) Voltage required to achieve full LCE contraction (~45%) using metallic wires of varying thickness. Metallic wires thinner than 100 μm exhibited insufficient mechanical robustness and frequent failure due to overheating, while wires thicker than 250 μm were too stiff and prone to mechanical fracture. The 150 μm-thick wire was selected as the optimal candidate, offering a balance between low voltage operation (<5 V), reliable actuation, and mechanical compliance. (**B**) Surface temperature profiles of the heating element under increasing current.

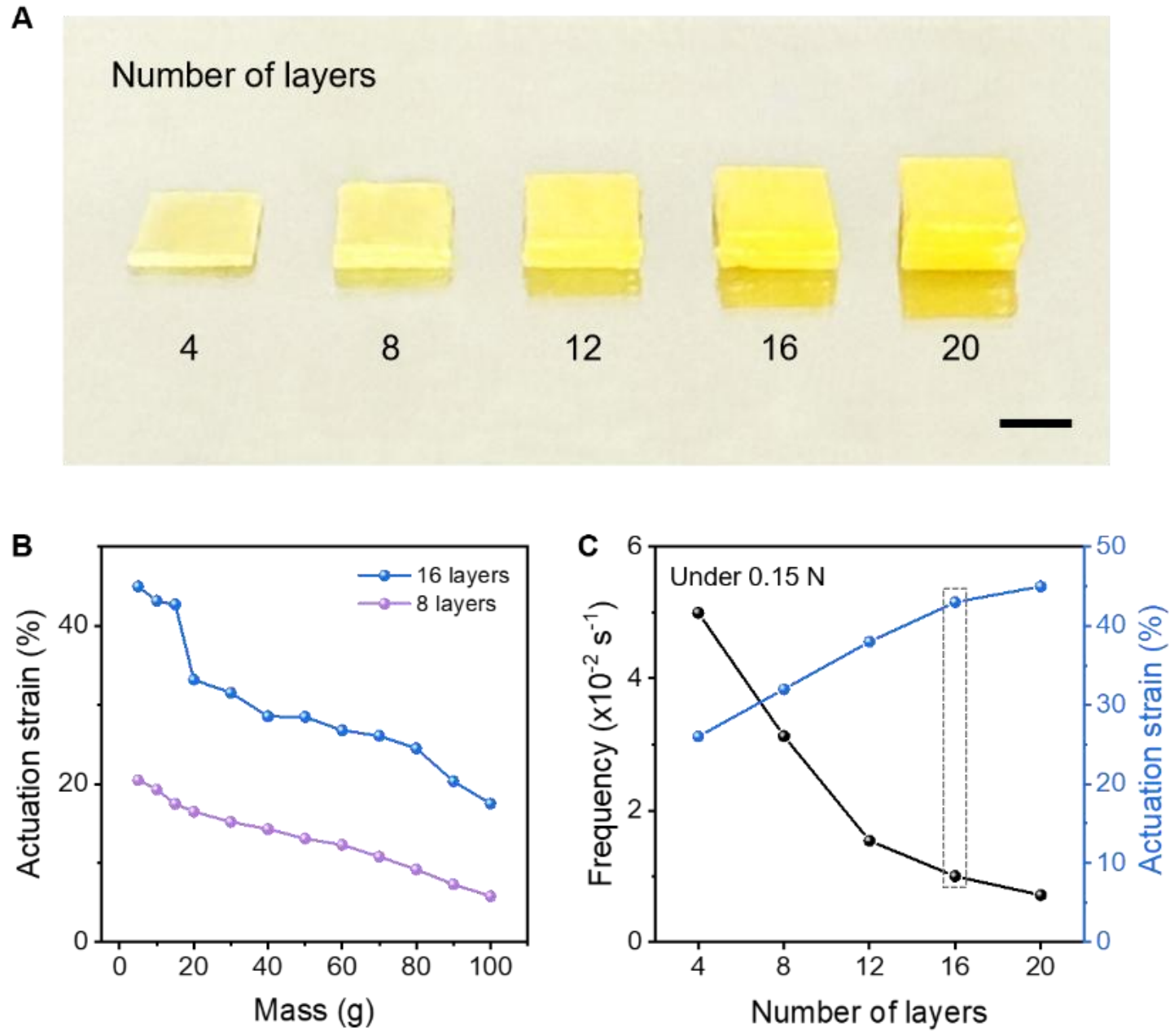

Fig. S6. Optimization of LCE actuator thickness for effective robot operation. (**A**) Optical images of LCE muscle modules fabricated with different layer numbers (4, 8, 12, 16, and 20) to tune actuator performance. Scale bar, 3 mm. (**B**) Load-bearing capability test demonstrating the contraction performance of LCE actuators with different thicknesses under external loading conditions. (**C**) Actuation frequency and strain under a 0.15 N load as functions of LCE layer count.

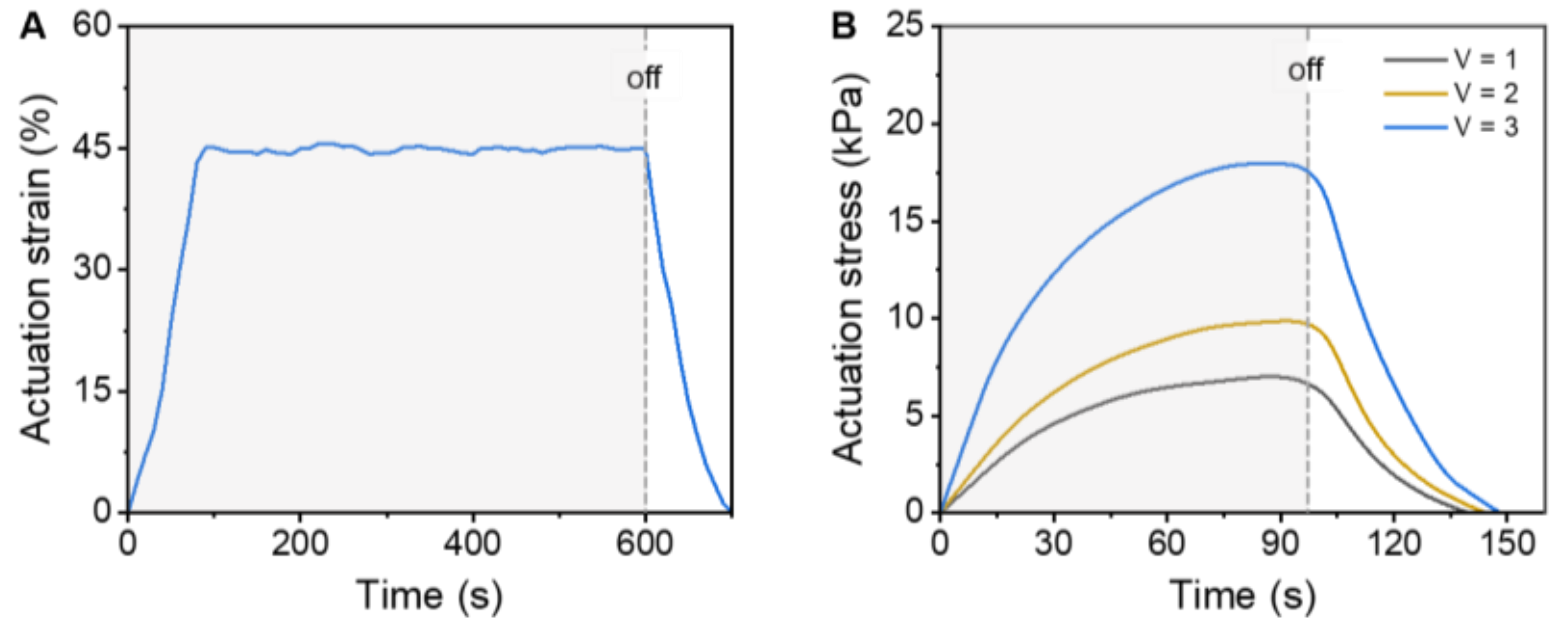

Fig. S7. Electrothermal actuation performance of the muscle modules. (**A**) Time-resolved strain profile of an LCE muscle under a constant voltage (3 V) over a continuous 10 min operation period. (**B**) Actuation stress-time curves recorded under applied voltages of 1, 2, and 3 V.

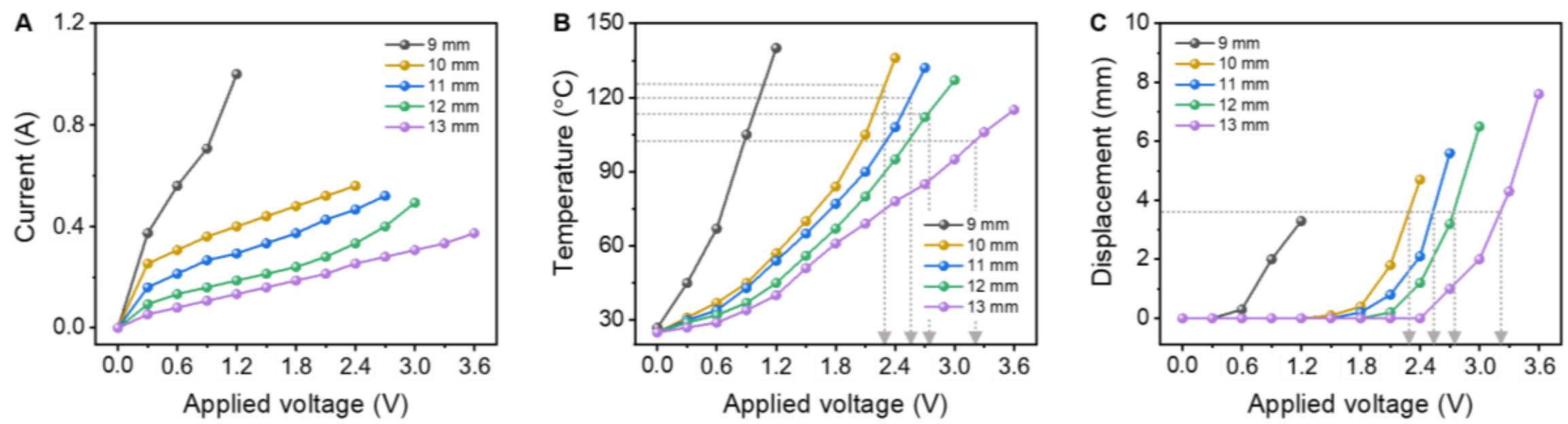

Fig. S8. Length-dependent electrothermal actuation behavior of artificial muscle modules. (**A**) Electrical current as a function of applied voltage for LCE muscle modules of varying lengths. (**B**) Surface temperature–voltage profiles for different muscle lengths. (**C**) Voltage-dependent displacement characteristics of each muscle length.

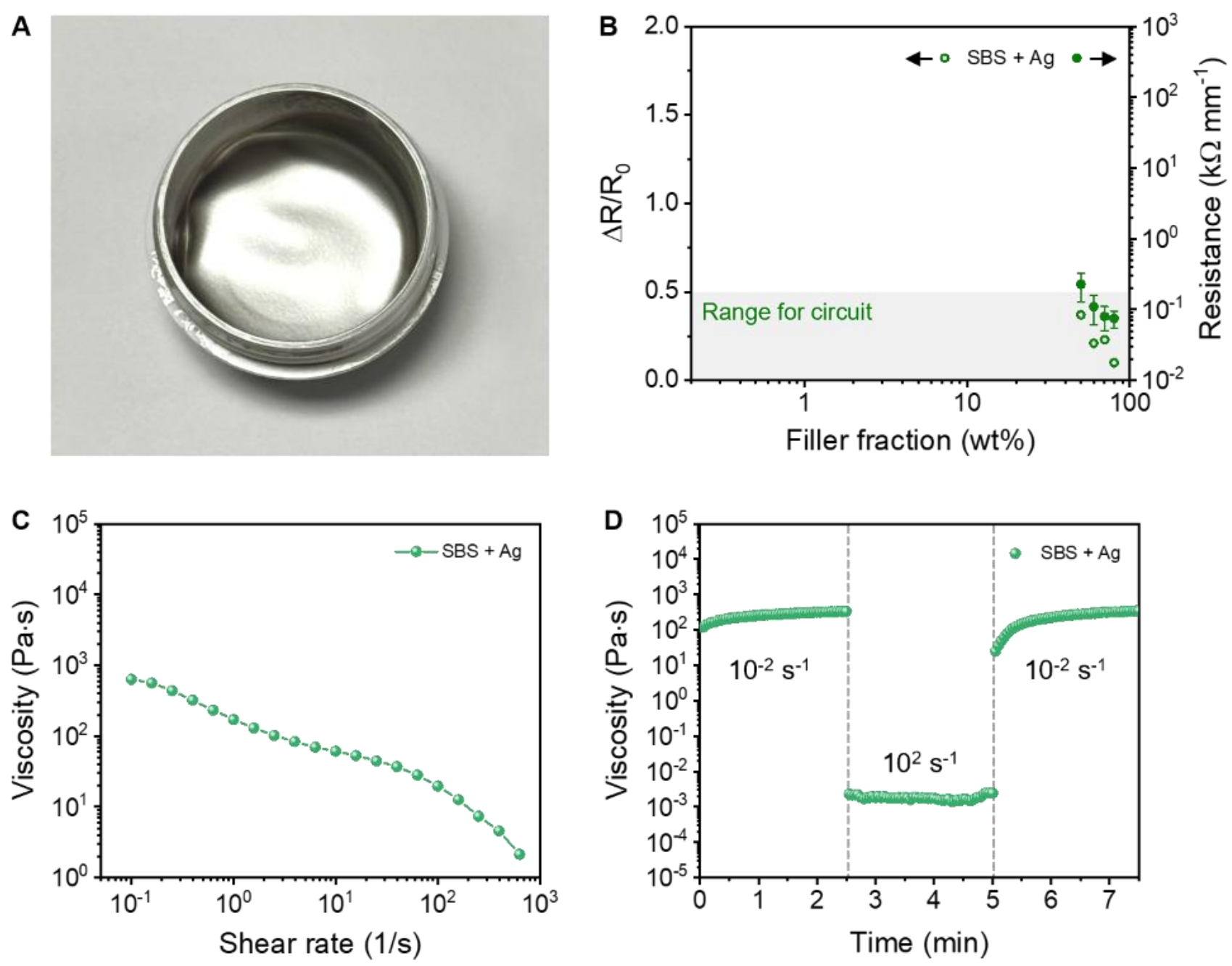

Fig. S9. Optimization and rheological characterization of conductive ink for flexible circuit interconnects. (**A**) Optical image of SBS-based conductive ink containing 70 wt% silver filler. (**B**) Relative resistance change of printed lines as a function of silver filler loading, revealing a percolation threshold and identifying 70 wt% as an optimal balance between electrical conductivity and mechanical compliance. (**C**) Shear rate-dependent viscosity profile of the conductive ink. (**D**) Time-resolved viscosity measurements under stepwise shear rate variation.

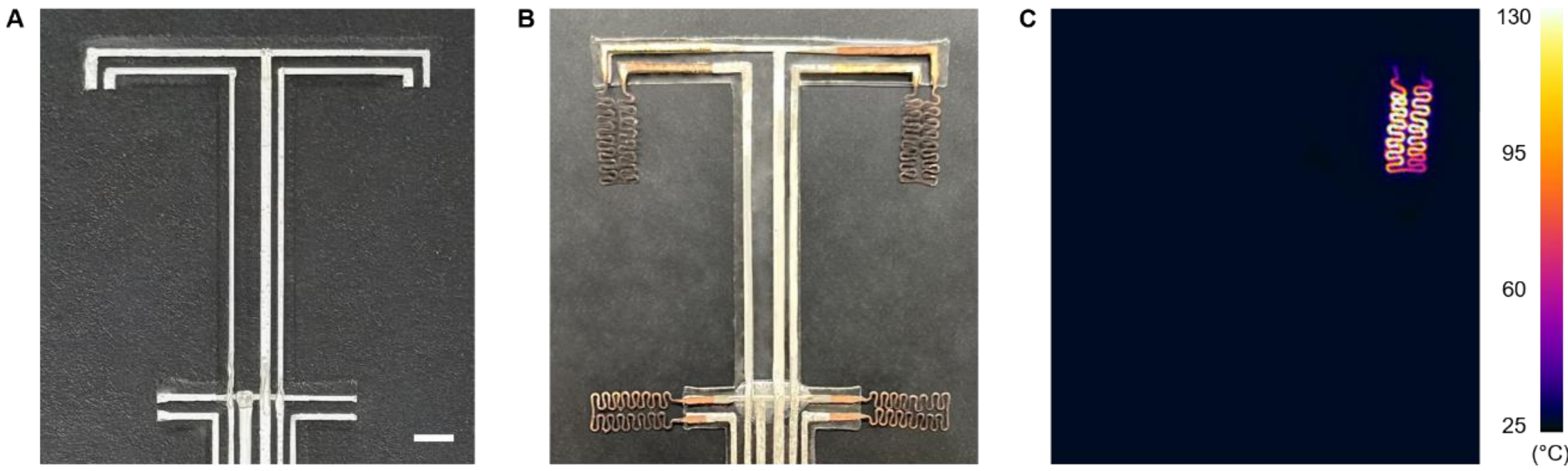

Fig. S10. Integration and heating performance of a flexible polymeric circuit for selective thermal actuation. (**A**) Optical image of the fully printed flexible polymeric circuit, designed with multiple independent traces for spatially addressable actuation. Scale bar, 5 mm. (**B**) Optical image showing the integration of heating elements at the circuit ends, enabling electrothermal actuation of LCE muscle modules. (**C**) Infrared thermal image demonstrating localized and selective heating at specific regions under electrical stimulation.

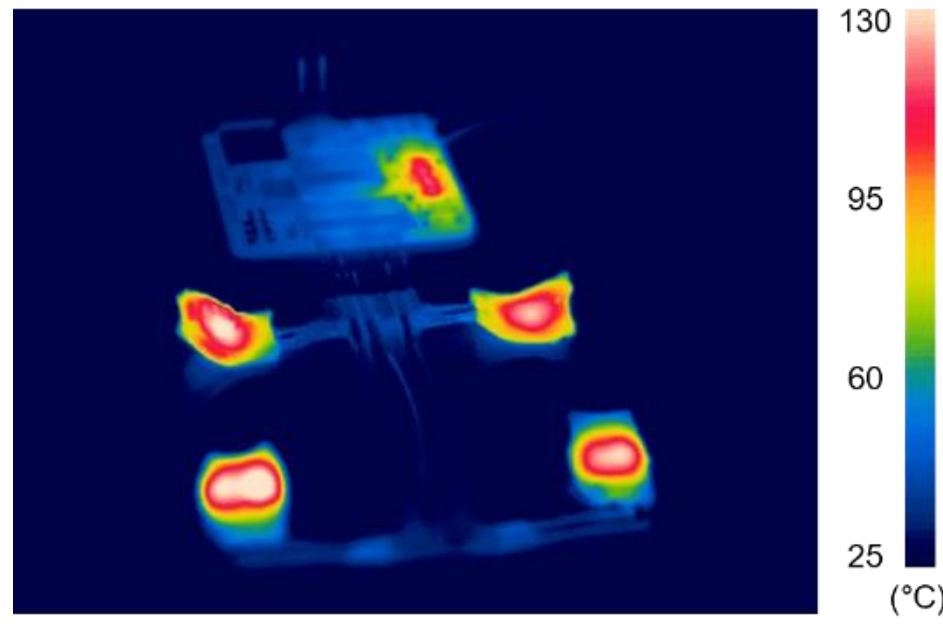

Fig. S11. Independent and simultaneous activation of modular muscle components. Infrared thermal image of the wearable muscle platform demonstrating spatially controlled activation. Localized heating patterns confirm the selective and concurrent electrothermal stimulation of all embedded muscle modules. Color scale indicates surface temperature distribution (25–130 °C).

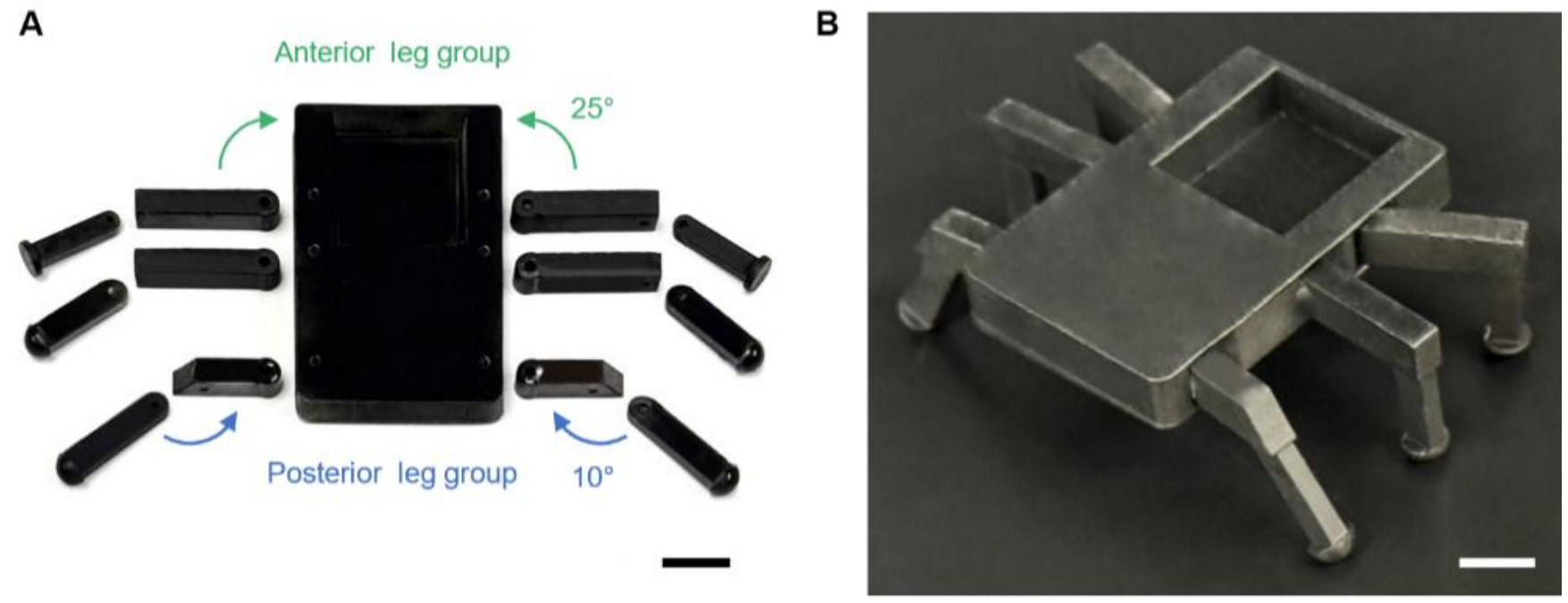

Fig. S12. Design and assembly of the 3D printed beetle-inspired robot frame. (**A**) Layout of individual 3D-printed segments including the main body and six modular legs, grouped into anterior and posterior sets designed for active motion. Scale bar, 1 cm. (**B**) Assembled frame constructed using interlocking hole-and-pillar joints, with anterior and posterior leg groups left unfixed to enable programmable motion while the middle legs remain static for support. Scale bar, 1 cm.

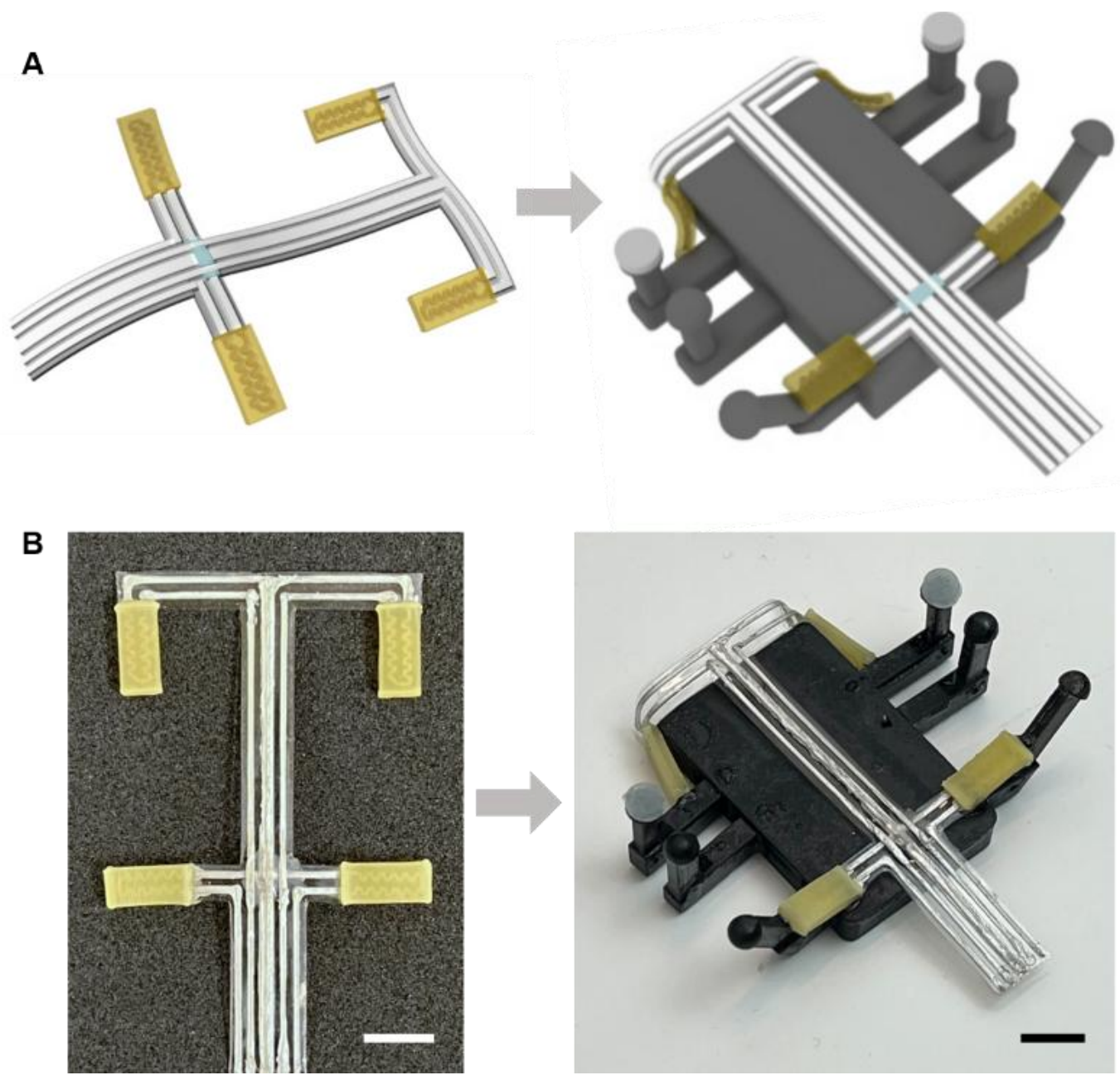

Fig. S13. Assembly of the soft-actuated beetle-inspired robot. (**A**) Schematic illustration of the module components attached to the robot frame. Scale bar, 1 cm. (**B**) Fully assembled robot integrating the soft actuation modules and footpads. Scale bar, 1 cm.

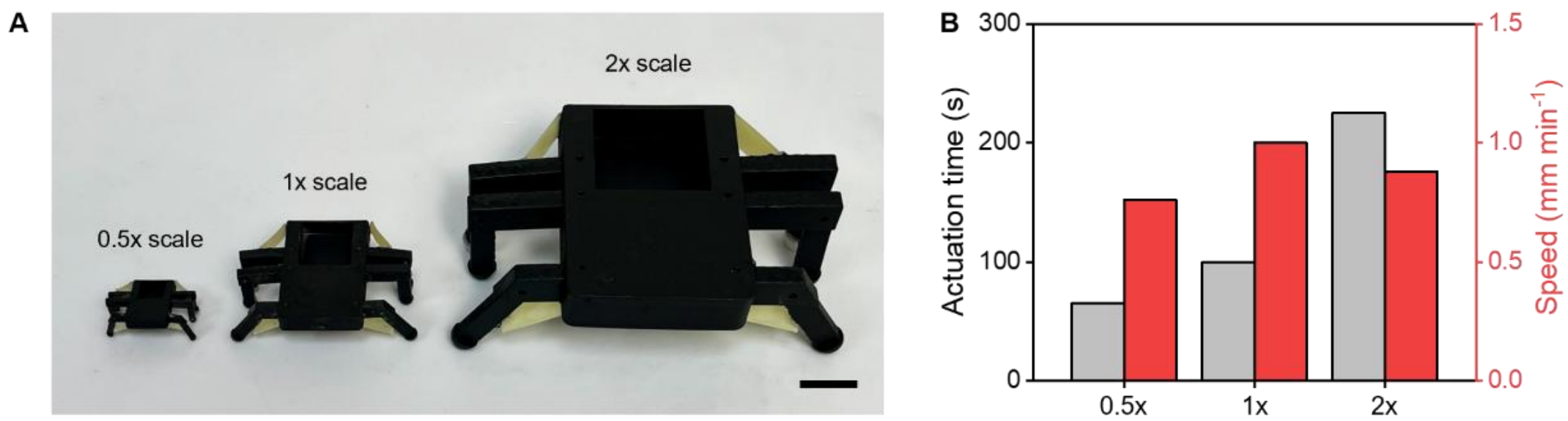

Fig. S14. Scalability of the soft robotic platform enabled by additive manufacturing. (**A**) Optical images of assembled robots fabricated at 0.5×, 1×, and 2× scales using the same 3D printing methodology for both frame and actuation modules. Scale bar, 2 cm. (**B**) Quantitative comparison of muscle actuation time and simulated walking speed across different robot sizes.

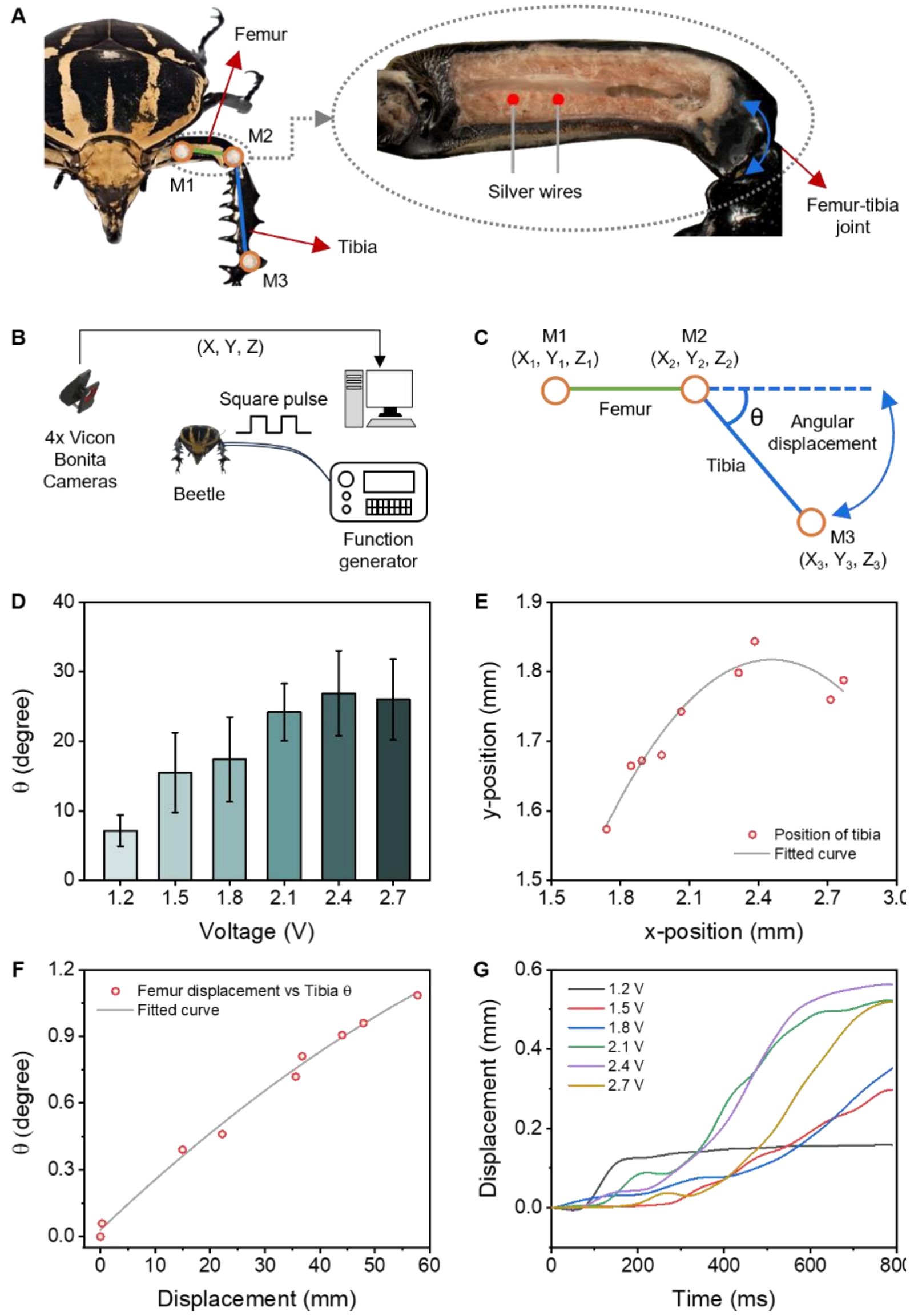

Fig. S15. Quantitative analysis of beetle foreleg muscle actuation to inform robotic design. (**A**) Anatomical mapping and marker placement on the femur and tibia segments of a *Mecynorrhina torquata* beetle for motion tracking. Reflective markers (Ø 3 mm) were positioned to measure joint kinematics, with electrode implantation and exposed muscle area indicated. (**B**) Schematic of the experimental setup utilizing a VICON motion capture system and electrical stimulator to record muscle-induced tibial motion. (**C**) Calculation of joint angle (θ) from marker positions to quantify tibia movement under stimulation. (**D**) Angular displacement of the tibia as a function of increasing stimulation voltages (1.2–2.7 V). (**E**) Conversion of angular to linear displacement based on geometric analysis of the tracked trajectory. (**F**) Regression curve correlating tibial angle and muscle contraction displacement. (**G**) Voltage-to-displacement calibration curve, confirming consistent actuation trends.

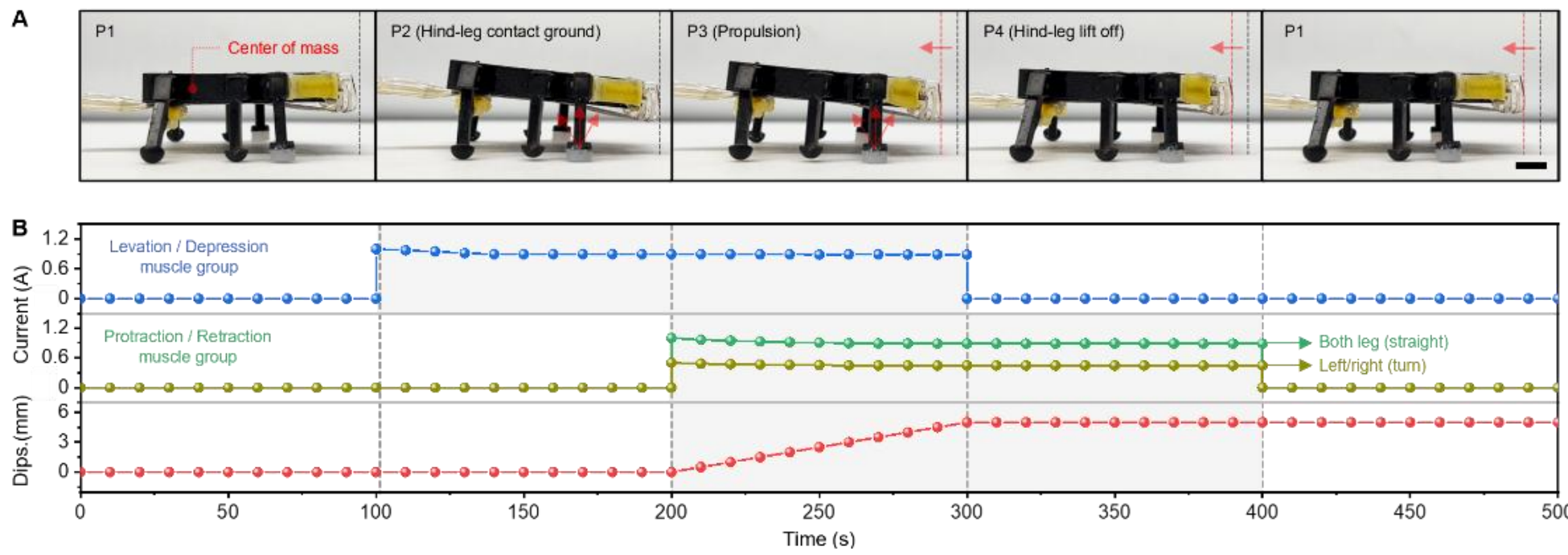

Fig. S16. Bioinspired locomotion mechanism of the robot. (**A**) Time-sequenced optical images illustrating the four phases of a single gait cycle: P1 (neutral stance), P2 (anterior leg actuation with hind leg contact), P3 (posterior leg actuation generating forward propulsion), and P4 (hind leg lift-off returning to P1). The center of mass is designed to shift dynamically to maintain balance and forward movement. Scale bar, 1 cm. (**B**) Synchronized current inputs to the anterior and posterior actuator groups and corresponding displacement of the robot body during each phase. The cyclic activation pattern confirms functional coordination between leg groups, mimicking the alternating muscle activation observed in biological insect gaits.

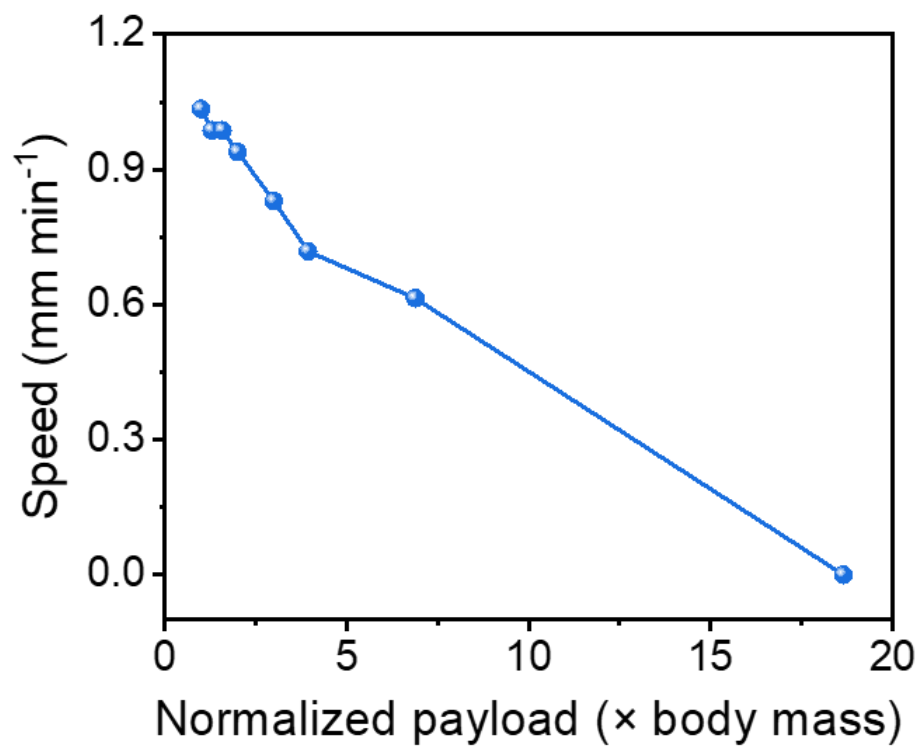

Fig. S17. Payload-dependent locomotion performance of BeetleBot. Locomotion speed as a function of normalized payload mounted on the robot body. Payload masses of 0, 5, 10, 17, 34, 50, 100, and 300 g were normalized to the robot body mass (17 g).

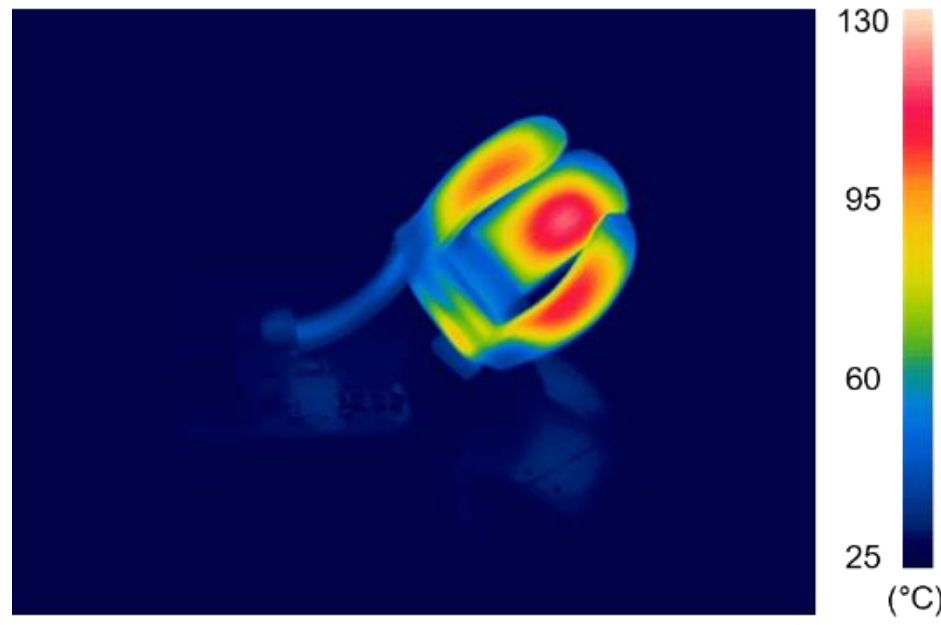

Fig. S18. Infrared visualization of electrothermal actuation in the soft gripper module. Thermal image showing the localized temperature distribution across the LCE-based gripper during electrical stimulation. The temperature increase near the embedded heater regions induces asymmetric contraction, resulting in controlled bending motion of the soft actuator. The thermal gradient highlights spatially selective activation essential for precise gripping tasks. Color scale indicates surface temperature distribution (25–130 °C).

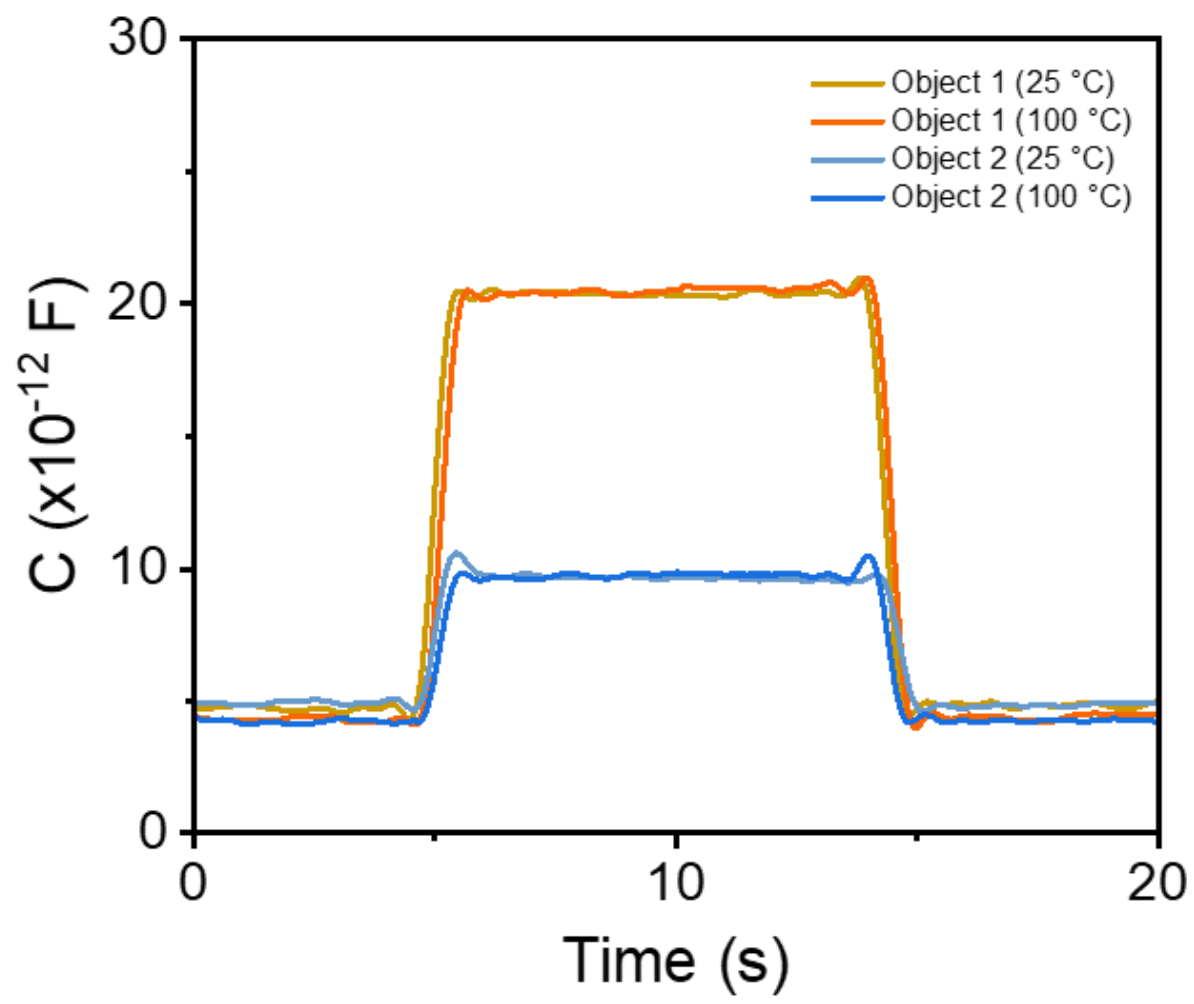

Fig. S19. Thermal stability of integrated capacitive pressure sensors during electrothermal actuation. Thermal stability characterization of the integrated capacitive pressure sensors under room temperature (25 °C) and elevated temperature conditions (100 °C), corresponding to the operating temperature range of the neighboring LCE actuator. The nearly identical baseline capacitance and sensing amplitudes observed under both conditions demonstrate minimal thermal interference and stable sensor performance during electrothermal actuation.

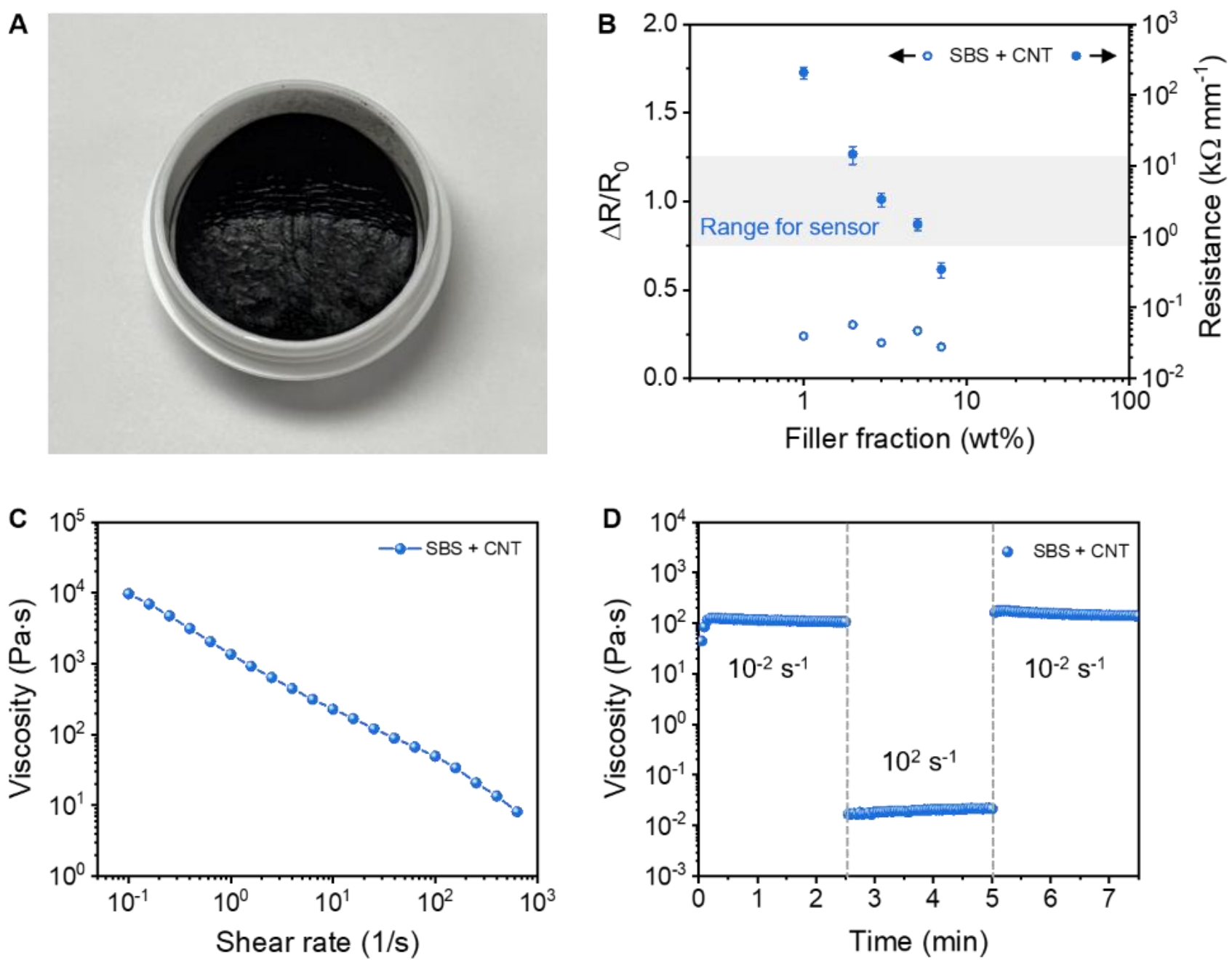

Fig. S20. Electrical performance and rheological properties of printable CNT-SBS composite inks for reliable strain sensing. (**A**) Optical image of a homogeneous CNT-SBS ink containing 5 wt% CNT filler content. (**B**) Relative resistance change of the composite ink as a function of CNT loading, identifying the optimal range for achieving both sufficient conductivity and resistance variation suitable for stretchable sensing. (**C**) Shear rate-dependent viscosity profile of composite ink. (**D**) Viscosity variations under varying shear rates, confirming its suitability for printing.

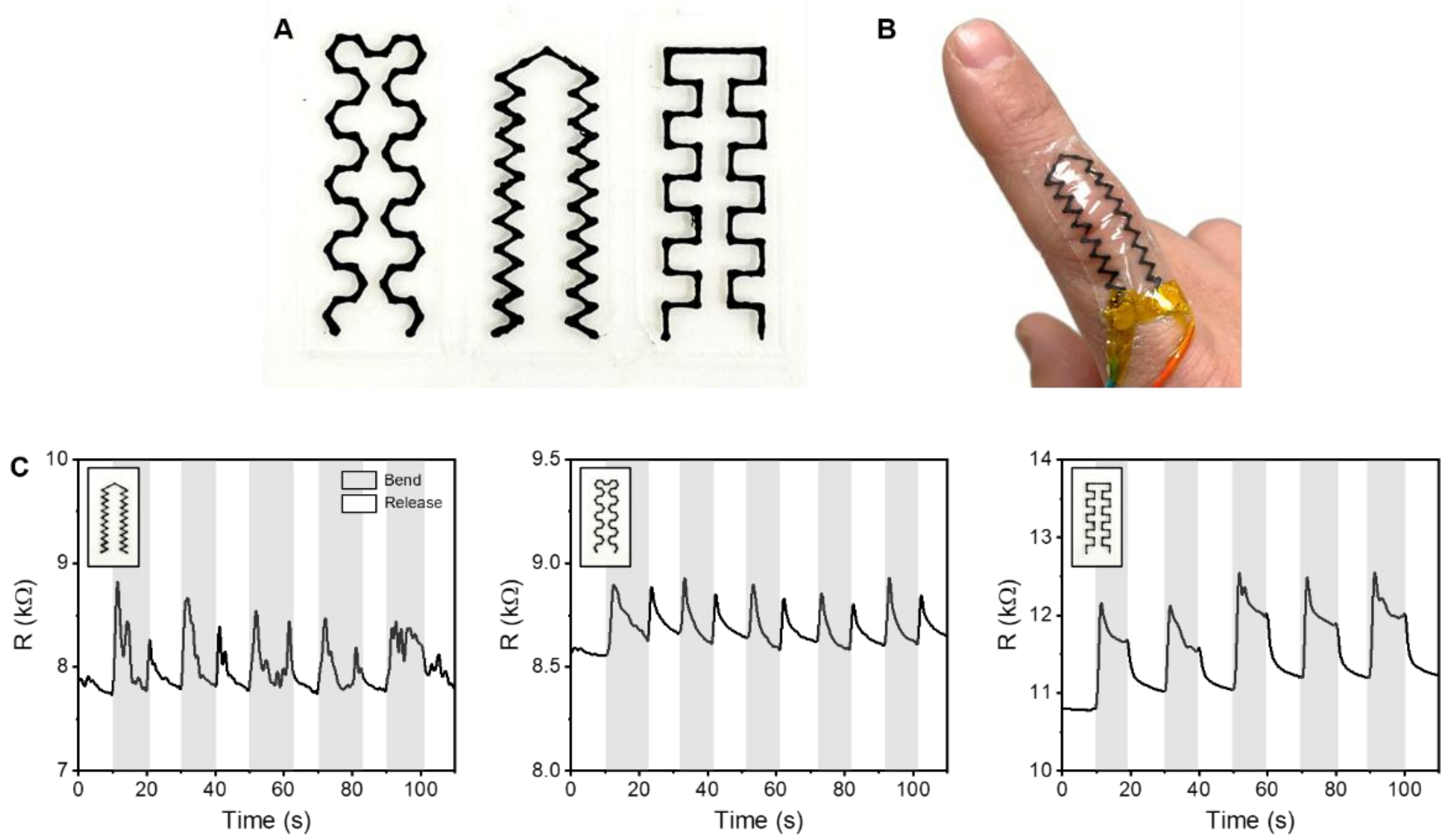

Fig. S21. Geometry optimization of wearable strain sensors for human gesture sensing. (**A**) Optical images of strain sensor patterns with different structural geometries, including serpentine, zigzag, and linear-interconnected designs fabricated using 3 wt% CNT-loaded SBS composite ink. (**B**) Photograph showing conformal attachment of the wearable strain sensor on a user's finger. (**C**) Resistance responses of the different sensor geometries under repeated finger bending and release cycles.

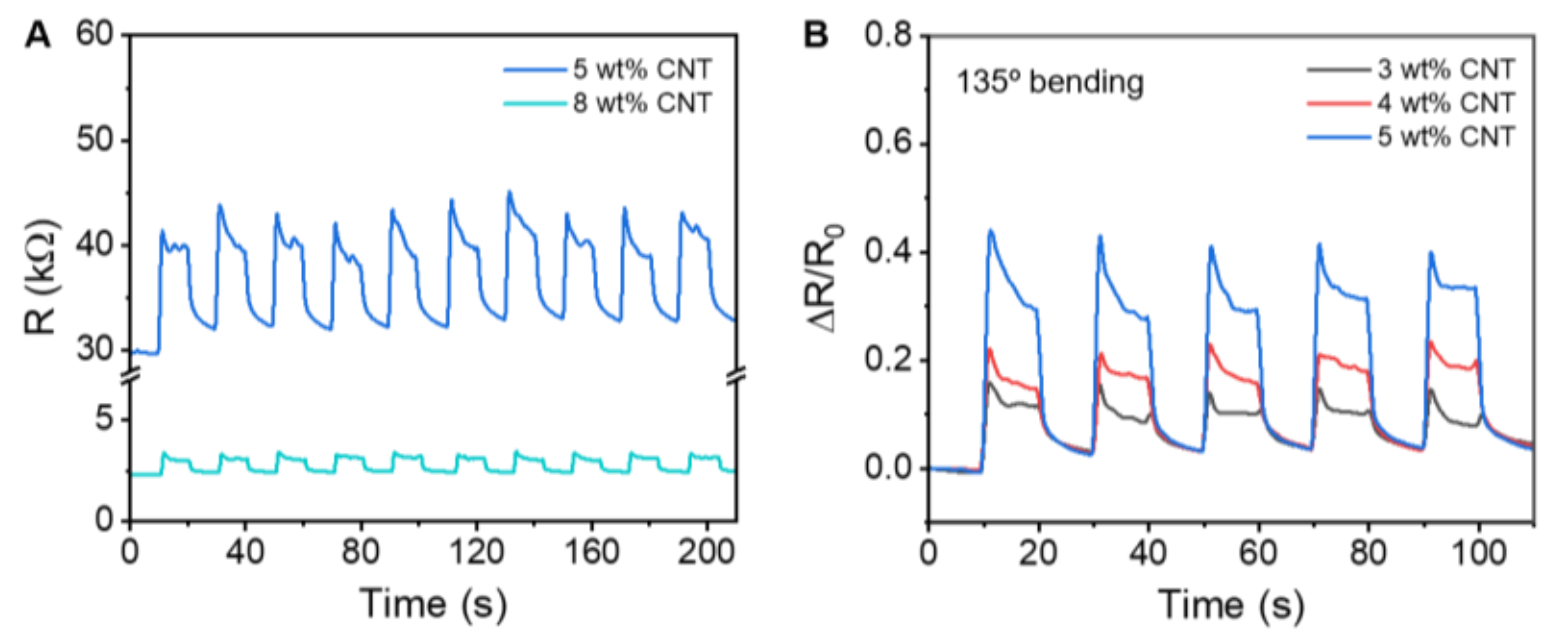

Fig. S22. Optimization of CNT filler ratio in CNT-SBS composites for strain sensing performance. (**A**) Resistance range of CNT-SBS composites with varying CNT concentrations under repeated 135° bending cycles, indicating that concentrations above 5 wt% yield reduced sensitivity and signal saturation. (**B**) Resistance variation profiles of composites with low CNT concentrations, verifying that 5 wt% is the optimal loading to achieve a broad dynamic range and high strain sensitivity for reliable sensor operation.

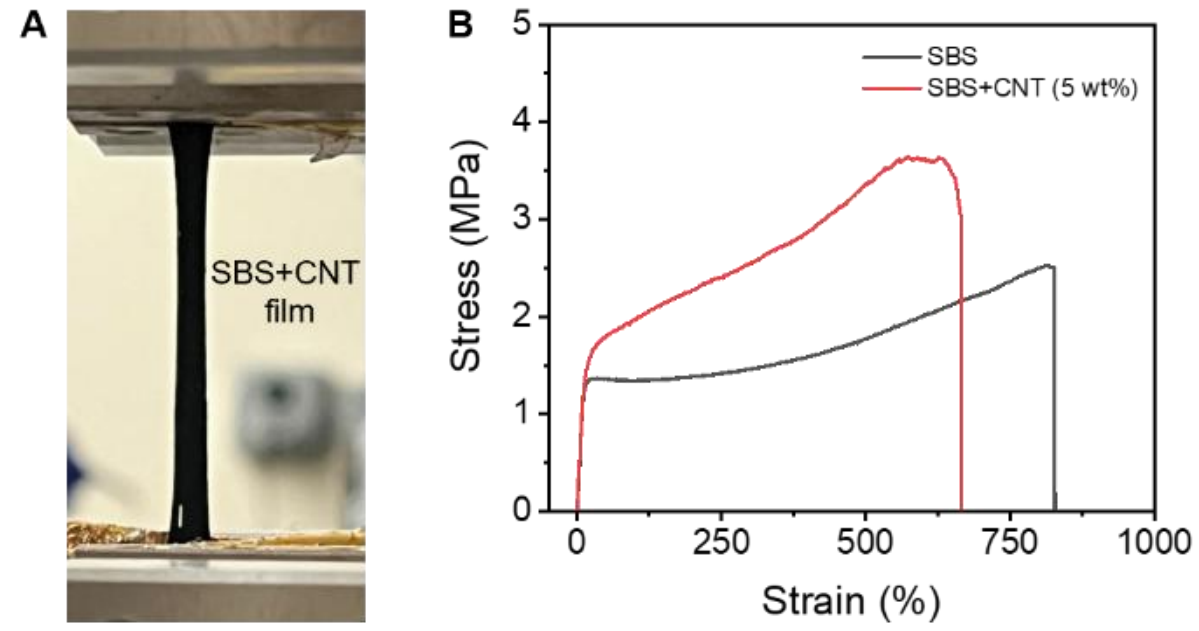

Fig. S23. Mechanical performance of CNT-SBS composite for strain sensor integration. (**A**) Optical image of the CNT-SBS composite film with 5 wt% CNT content undergoing uniaxial tensile testing. (**B**) Stress–strain curves comparing the mechanical behavior of pristine SBS and 5 wt% CNT-SBS composite films, demonstrating that the composite maintains high stretchability and mechanical robustness suitable for wearable strain sensing applications.

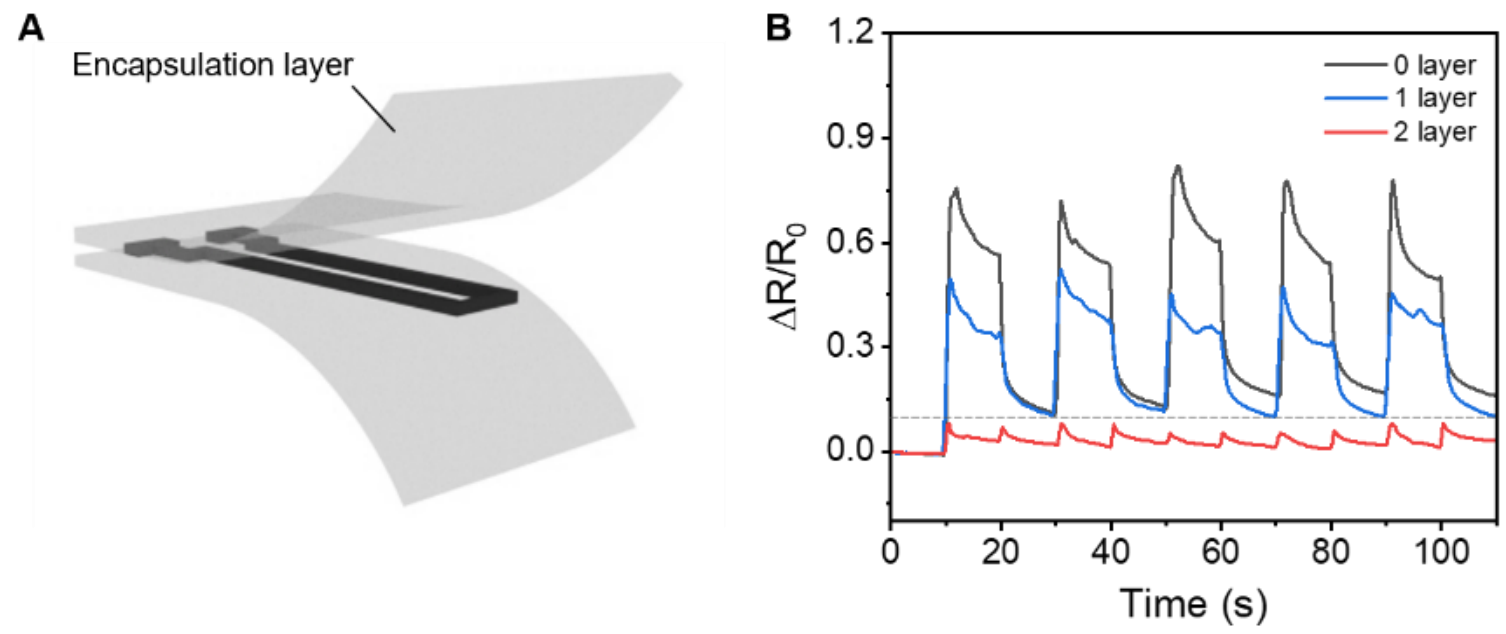

Fig. S24. Structural optimization of the strain sensor for mechanical reliability and signal stability. (**A**) Schematic illustration of the strain sensor structure, composed of a flexible substrate layer, a patterned sensing layer, and one or more additional encapsulation layers. (**B**) Resistance response during cyclic bending tests for sensors with 0, 1, and 2 encapsulation layers. Devices with two encapsulation layers exhibit reduced sensitivity due to increased modulus, while unencapsulated devices display unstable baseline resistance, confirming the necessity of a single encapsulation layer for optimal performance.

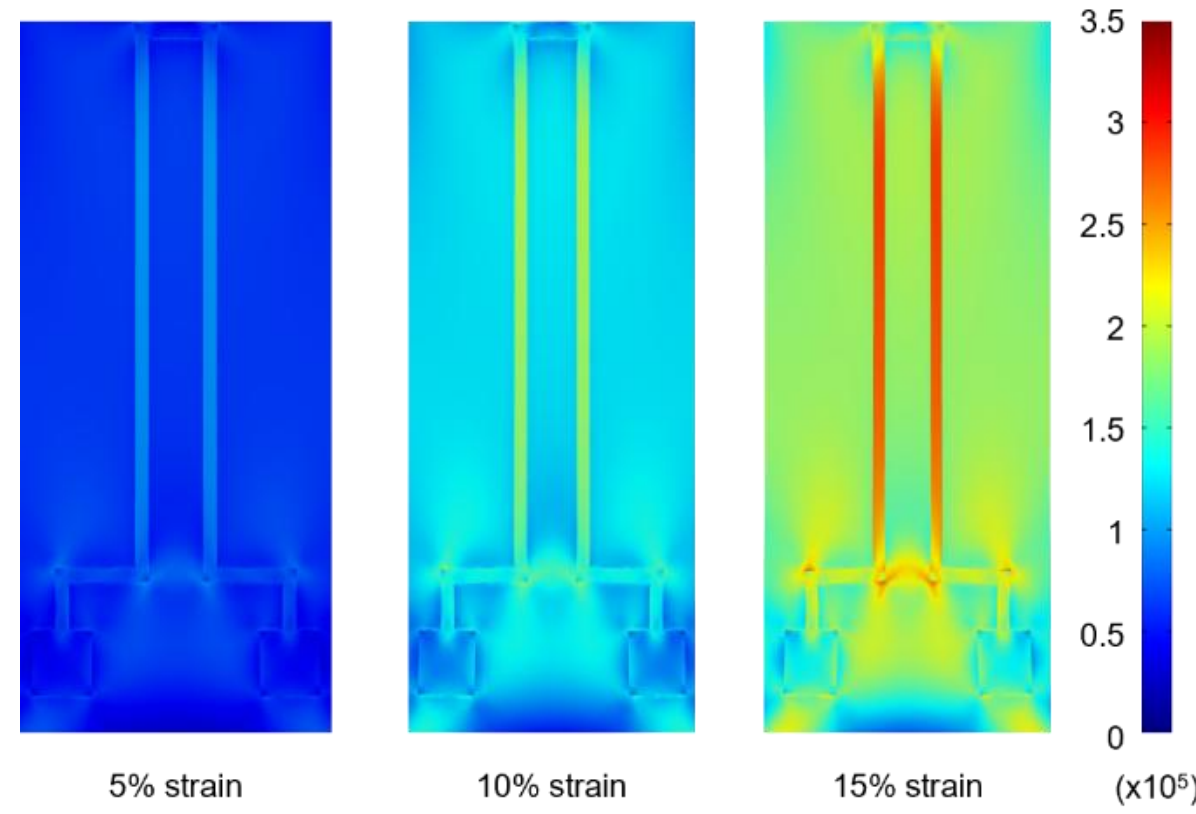

Fig. S25. Finite element analysis (FEA) of mechanical stress distribution in CNT-SBS strain sensors. FEA simulations depicting stress concentration profiles within printed CNT-SBS composite strain sensors under applied uniaxial strains of 5%, 10%, and 15%.

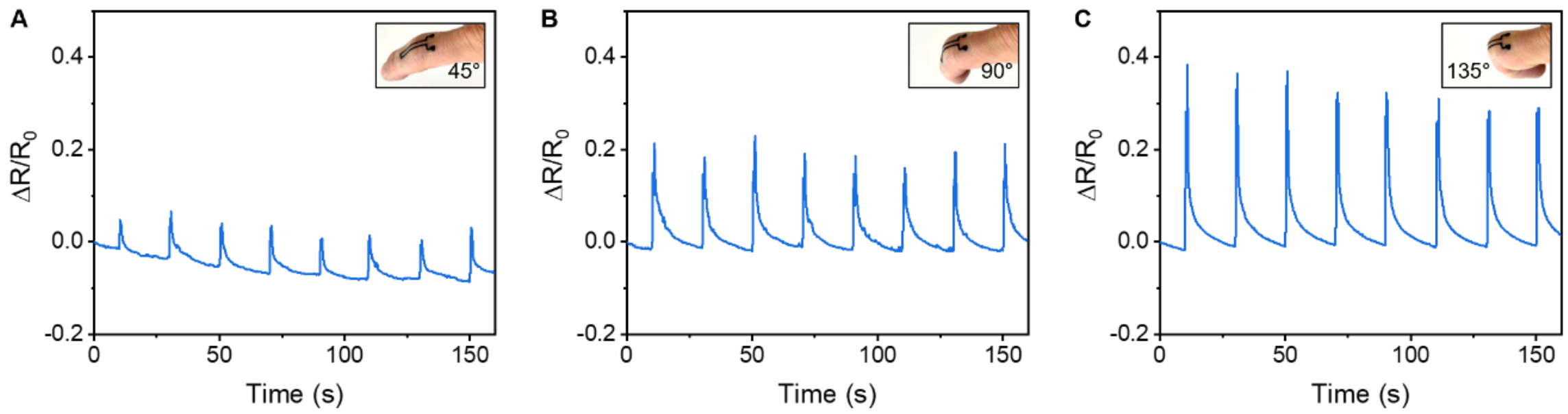

Fig. S26. Evaluation of strain sensor performance during finger bending. (**A** to **C**) Real-time resistance changes of the CNT-SBS-based strain sensor mounted on a user's finger under repeated bending at angles of 45°, 90°, and 135°.

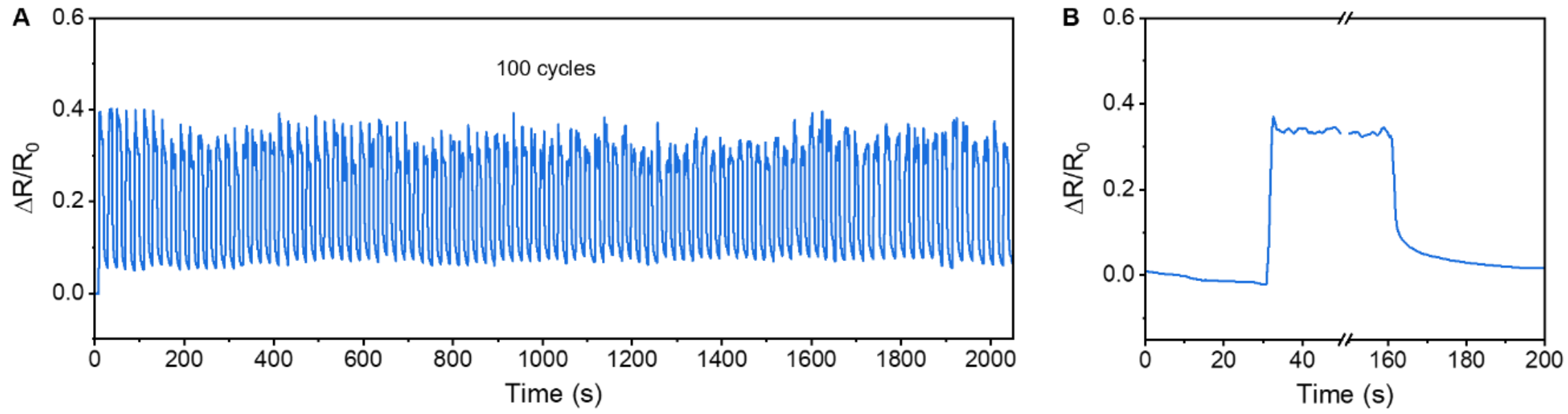

Fig. S27. Long-term stability and durability of the strain sensor during repetitive finger motion. (**A**) Electrical signal traces from the CNT-SBS strain sensor mounted on a finger of user during repeated 135° bending over 100 continuous cycle. (**B**) Stable resistance output maintained during prolonged finger bending for more than 2 min.

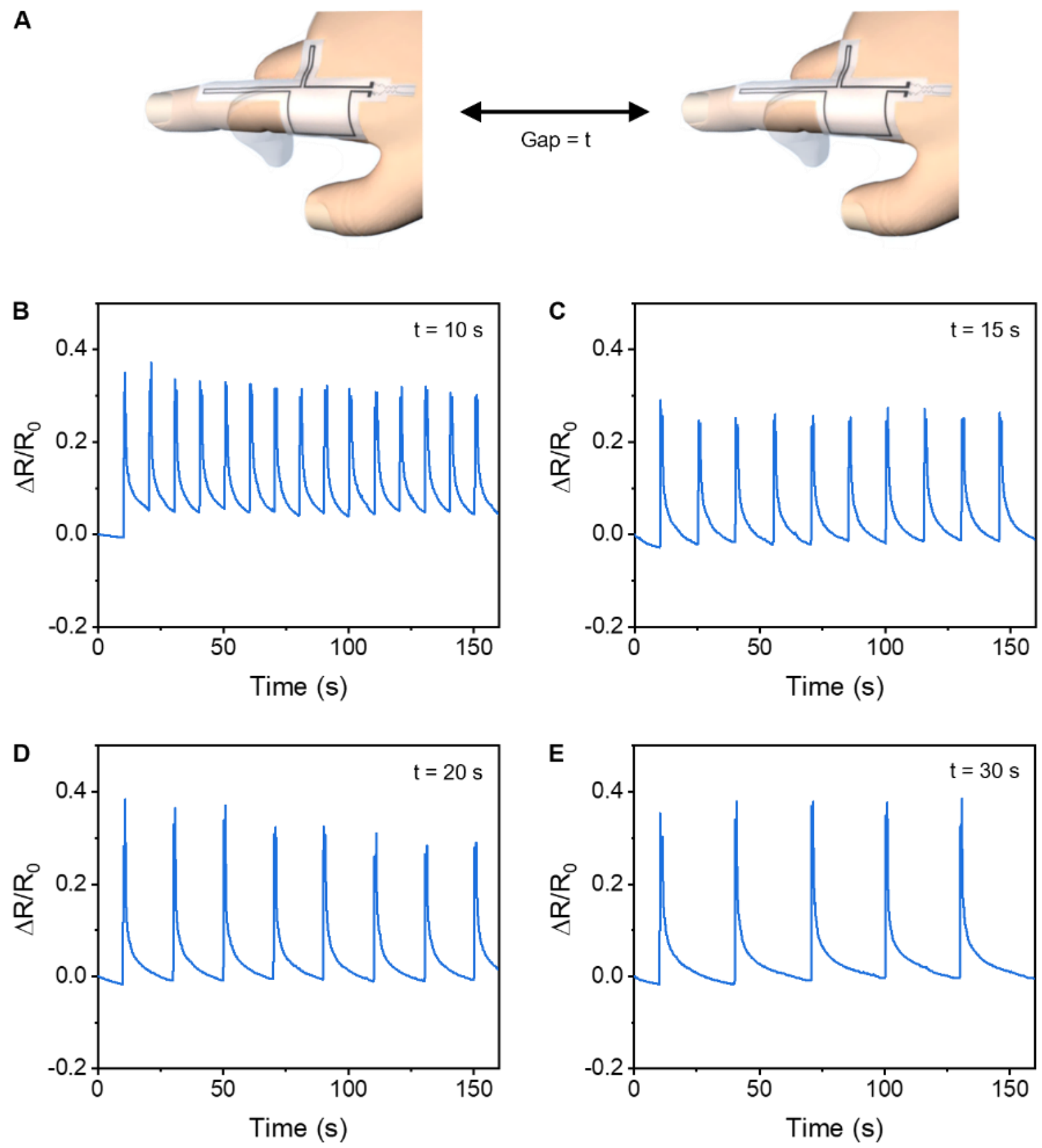

Fig. S28. Evaluation of temporal response consistency of the wearable finger patch under varying gesture intervals. (**A**) Schematic illustration of the experimental setup, illustrating the finger bending gesture and the interval between each actuation. (**B** to **E**) Resistance responses recorded at time intervals of 10, 15, 20, and 30 seconds, respectively, demonstrating that the optimized CNT-SBS strain sensor consistently tracks finger movements with minimal drift or delay, regardless of the temporal spacing between gestures.

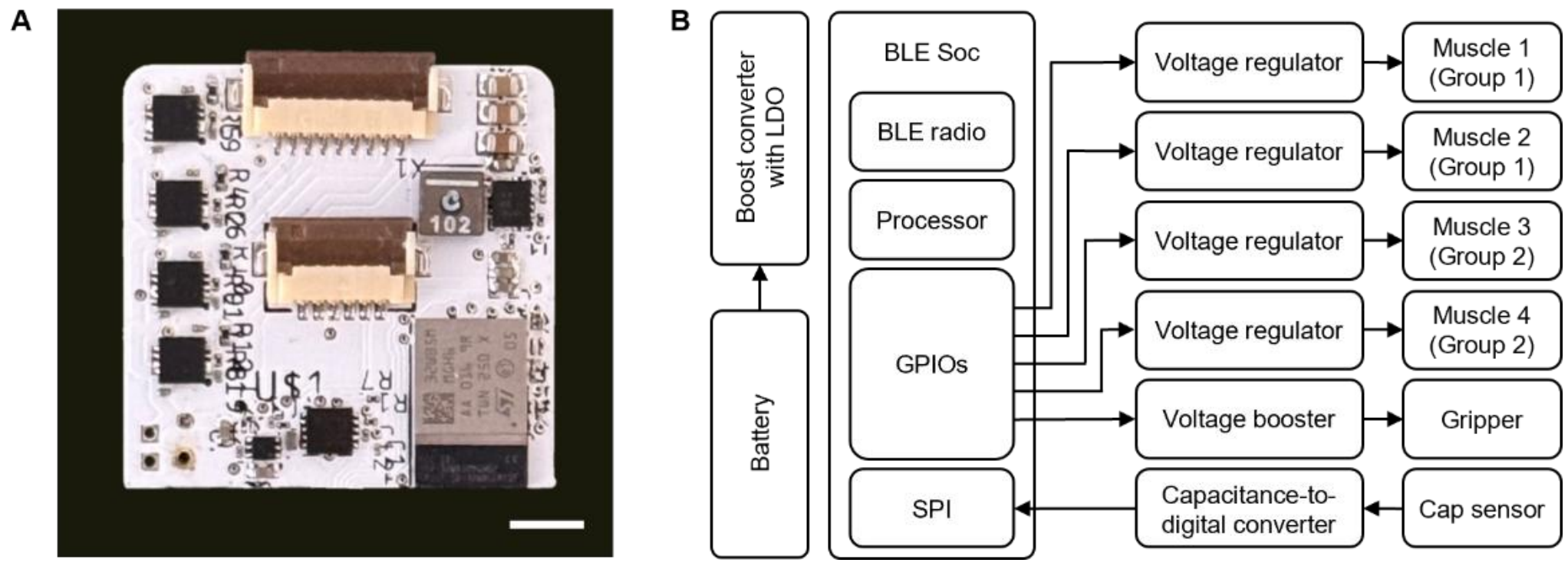

Fig. S29. Custom-designed circuit board for closed-loop robotic actuation and bidirectional human–machine interfacing. (**A**) Optical image of the fully assembled circuit board, dimensionally tailored to align with the robot's width for seamless integration. Scale bar, 5 mm. (**B**) Schematic layout of the printed circuit board illustrating component placement and electrical routing. (**C**) Block diagram depicting the signal flow between key functional modules.

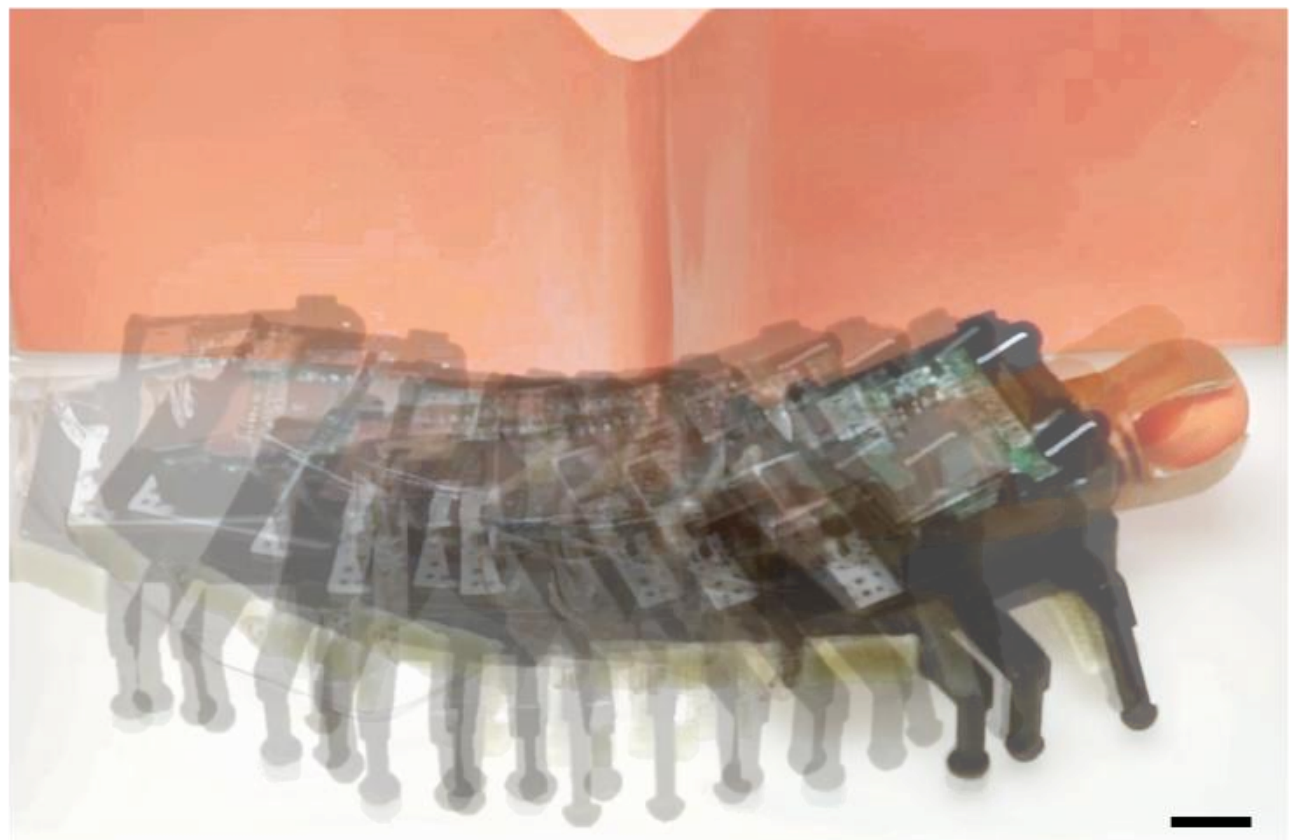

Fig. S30. Time-lapse visualization of soft robot navigating complex terrain. Sequentially overlaid optical images demonstrate the locomotion of the soft robot as it autonomously performs navigation and object sensing on behalf of the user across unstructured terrain. Scale bar, 1 cm.

Table S1. Comparison of BeetleBot with representative soft robotic systems. Comparison of representative soft robotic platforms across key functional categories, including actuation mechanism, selective actuation, locomotion capability, object manipulation, physical sensing, vision feedback, wireless control, closed-loop interaction, and representative performance features. O, demonstrated in the integrated system; Δ, partially demonstrated or externally assisted; X, not reported or not applicable.

**Robotic systems**	**Actuation mechanism**	**Selective actuation**	**Locomotion modes**	**Object manipulation**	**Physical sensing**	**Vision feedback**	**Wireless control**	**Closed-loop feedback**	**Performance Feature**	**Ref.**
Multigait soft robot	Pneumatic actuation	Δ	Crawling, undulation, obstacle navigation	X	X	X	X	Open-loop control	Multiple gaits, low-pressure pneumatic operation	(*28*)
Octobot	Pneumatic actuation	Δ	Cyclic arm actuation	X	X	X	X	Autonomous microfluidic logic	Entirely soft autonomous operation	(*29*)
SMA soft robot	Shape-memory polymer	O	Crawling / dynamic locomotion	X	X	X	O	Open-loop gait control	Untethered locomotion	(*30*)
Soft robotic fish	Hydraulic actuation	O	3D swimming	X	X	O	O	Human-supervised operation	Swimming, continuous visual recording	(*31*)
LEAP soft robot	Pneumatic actuation	Δ	Galloping, swimming	O	X	X	X	Open-loop actuation	High-speed locomotion, high-force gripping	(*32*)
Soft-legged robot	Pneumatic actuation	O	Walking gait	X	Δ	O	O	Embodied pneumatic gait control	Gait generation without electronic components	(*33*)
Self-powered soft robot	Dielectric elastomer actuation	Δ	Swimming	X	X	Δ	Δ	Onboard power/control locomotion	Operation under hydrostatic pressure	(*34*)
Soft octopus arm	Pneumatic actuation	O	Arm reaching motion	O	O	X	Δ	Sensory feedback interaction	Reaching, sensing, grasping, interacting	(*35*)
BeetleBot	LCE electrothermal actuation	O	Forward walking, left/right turning	O	O	O	O	Human-in-the-loop visual/tactile feedback	Low-voltage actuation, integrated locomotion, manipulation, sensing, HMI	This work

Movie legends

Movie S1. Multicycle electrothermal actuation of a single LCE muscle module.

Movie S2. Fabrication process of a flexible polymeric circuit via direct ink writing.

Movie S3. Sequential printing of top and bottom LCE muscle layers onto the flexible circuit.

Movie S4. Independent electrical activation of muscle groups within the wearable muscle platform.

Movie S5. Direct ink writing-based fabrication of a wearable finger patch controller.

Movie S6. Object detection by the soft gripper.

Movie S7. Real-time gesture-controlled locomotion of the BeetleBot with simultaneous visual feedback.
